# Selective degradation of mutant FMS-like tyrosine kinase-3 requires BIM-dependent depletion of heat shock proteins

**DOI:** 10.1038/s41375-024-02405-5

**Published:** 2024-09-17

**Authors:** Melisa Halilovic, Mohamed Abdelsalam, Joanna Zabkiewicz, Michelle Lazenby, Caroline Alvares, Matthias Schmidt, Walburgis Brenner, Sara Najafi, Ina Oehme, Christoph Hieber, Yanira Zeyn, Matthias Bros, Wolfgang Sippl, Oliver H. Krämer

**Affiliations:** 1 Department of Toxicology, University Medical Center, 55131 Mainz, Germany; 2 Department of Pharmaceutical Chemistry, Faculty of Pharmacy, Alexandria University, Alexandria, Egypt; 3 Department of Medicinal Chemistry, Institute of Pharmacy, Martin-Luther-University of Halle-Wittenberg, Halle, Saale Germany; 4 Academic Department of Haematology, University of Cardiff, Heath Park, Cardiff UK; 5 Clinic for Obstetrics and Women’s Health, University Medical Center, 55131 Mainz, Germany; 6 Hopp Children’s Cancer Center Heidelberg (KiTZ), Heidelberg, Germany; 7 Clinical Cooperation Unit Pediatric Oncology (B310), German Cancer Research Center (DKFZ) and German Cancer Consortium (DKTK), Heidelberg, Germany; 8 National Center for Tumor Diseases Heidelberg, Heidelberg, Germany; 9 Department of Dermatology, University Medical Center Mainz, Mainz, Germany

**Keywords:** Acute myeloid leukaemia, Cell signalling, Apoptosis

## Abstract

Internal tandem duplications in the FMS-like tyrosine kinase-3 (FLT3-ITD) are common mutations in acute myeloid leukemia (AML). Proteolysis-targeting chimeras (PROTACs) that induce proteasomal degradation of mutated FLT3 emerge as innovative pharmacological approach. Molecular mechanisms that control targeted proteolysis beyond the ubiquitin-proteasome-system are undefined and PROTACs are the only known type of FLT3 degraders. We report that the von-Hippel-Lindau ubiquitin-ligase based FLT3 PROTAC MA49 (melotinib-49) and the FLT3 hydrophobic tagging molecule MA50 (halotinib-50) reduce endoplasmic reticulum-associated, oncogenic FLT3-ITD but spare FLT3. Nanomolar doses of MA49 and MA50 induce apoptosis of human leukemic cell lines and primary AML blasts with FLT3-ITD (*p* < 0.05-0.0001), but not of primary hematopoietic stem cells and differentiated immune cells, FLT3 wild-type cells, retinal cells, and c-KIT-dependent cells. In vivo activity of MA49 against FLT3-ITD-positive leukemia cells is verified in a *Danio rerio* model. The degrader-induced loss of FLT3-ITD involves the pro-apoptotic BH3-only protein BIM and a previously unidentified degrader-induced depletion of protein-folding chaperones. The expression levels of HSP90 and HSP110 correlate with reduced AML patient survival (*p* < 0.1) and HSP90, HSP110, and BIM are linked to the expression of FLT3 in primary AML cells (*p* < 0.01). HSP90 suppresses degrader-induced FLT3-ITD elimination and thereby establishes a mechanistically defined feed-back circuit.

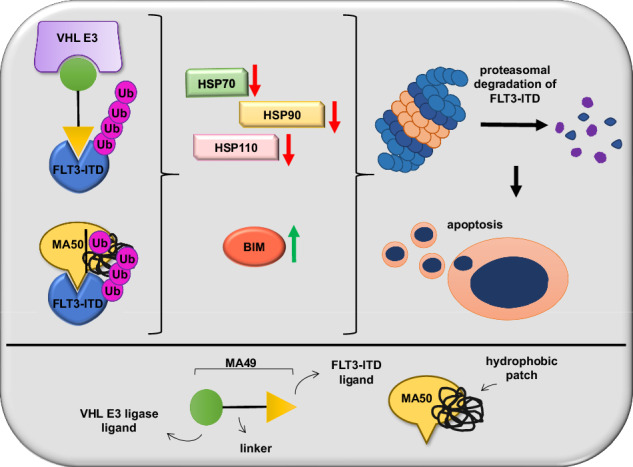

## Introduction

The *FLT3* gene encodes the class III receptor tyrosine kinase FMS-like tyrosine kinase-3 (FLT3), which is one of the main regulators of hematopoietic cell proliferation, survival, and differentiation. FLT3 is activated and autophosphorylated upon binding of the FLT3 ligand. This initiates FLT3 dimerization and subsequent activation of pro-proliferative PI3K/AKT and ERK/MAPK signaling pathways.

FLT3 is frequently hyperactivated in acute myeloid leukemia (AML) which represents one-third of leukemia cases worldwide. FLT3 mutants promote cancer cell proliferation and suppress the programmed cell death pathway of apoptosis through an overactivation of the above-named pro-proliferative signaling pathways and JAK/STAT5 signaling [1, 2]. The BCL2 protein family and enzymes of the caspase family control this non-inflammatory process [3]. According to the European Leukemia-Net classification, AML patients harboring FLT3 mutations are in the intermediate profile prognostic group. There are two types of activating FLT3 mutations, internal tandem duplications located in the juxtamembrane domain (FLT3-ITD) and point mutations in the FLT3 tyrosine kinase domain (FLT3-TKD). Whereas FLT3-ITD is an unfavorable mutation in AML and the proven cause of this cancer, FLT3-TKD is not linked to worse patients prognosis [4, 5].

First-generation FLT3 inhibitors, such as sunitinib, sorafenib, and midostaurin have low specificity. This can lead to undesired side-effects in patients [6–8]. To overcome such toxicological concerns, second-generation inhibitors, including quizartinib, crenolanib, and gilteritinib have been developed and clinically used to treat FLT3-mutated AML [9–11].

FLT3 inhibitors fall into type I and type II inhibitors based on their binding modes to FLT3. The conformation of FLT3 is determined by an aspartic acid-phenylalanine-glycine (Asp-Phe-Gly, DFG) stretch in the FLT3-TKD. Type I inhibitors (midostaurin, sunitinib, crenolanib, gilteritinib) bind to the active FLT3 conformation (“DFG-in”), while type II inhibitors (sorafenib, tandutinib, quizartinib) bind to FLT3 in its inactive conformation (“DFG-out”). Secondary mutations in the TKD of FLT3-ITD stabilize the active conformation. This prevents binding of type II inhibitors [10, 12]. Type I inhibitors remain active against TKD mutations in FLT3-ITD. However, some of these drugs inhibit the FLT3-related class kinase c-KIT. For example, the multi-kinase inhibitors midostaurin and crenolanib are stronger inhibitors of c-KIT than quizartinib. Simultaneous inhibition of FLT3 and c-KIT suppresses normal hematopoiesis. Gilteritinib only moderately inhibits c-KIT, but targets other kinases, such as LTK, AXL, and ALK, and is less potent against FLT3-ITD than quizartinib [7, 10, 13, 14].

Targeted protein degradation is a recent pharmacological approach in which proteins are eliminated through the ubiquitin-proteasome-system [15, 16]. Proteolysis-targeting chimeras (PROTACs) are heterobifunctional molecules containing ligands that bind target proteins, ligands that dock to E3 ubiquitin-ligases, and linkers connecting these moieties. The formation of a ternary complex induces poly-ubiquitination of the target protein which is recognized by 26S-proteasomes and rapidly degraded [15–17]. Further targeted protein degraders (TPDs) include hydrophobic tagging agents (HyTs). HyTs imitate exposed hydrophobic amino acids on protein surfaces. Chaperone proteins recognize such proteins as being misfolded. This triggers their poly-ubiquitination and proteasomal degradation. Key advantages of TPDs over low molecular inhibitors are the rapid and sustained elimination of their targets, high efficacy, and selectivity [15, 18].

A quizartinib-based FLT3 PROTAC that recruits the E3 ubiquitin ligase von-Hippel-Lindau-tumor suppressor (VHL) degrades ER-located and plasma membrane-bound FLT3. This PROTAC is like quizartinib less active against FLT3-ITD with TKD mutations. Although this PROTAC has fewer off-targets than quizartinib, it is a nanomolar c-KIT inhibitor [19]. A FLT3-PROTAC derived from the tyrosine kinase inhibitor (TKi) dovitinib and the cereblon (CRBN) E3 ligase subunit and a sunitinib-based VHL E3 ligase-recruiting FLT3 PROTAC eliminate FLT3-ITD and c-KIT [20, 21]. CRBN-based FLT3 PROTACs from modified quizartinib or gilteritinib have improved potency over their parent TKi [22–24]. A further FLT3 PROTAC is based on a purine inhibitor and additionally blocks the transcription regulator cyclin-dependent kinase-9 (ref. [25]). The activity of these PROTACs against AML cells with FLT3-ITD encourages the design and testing of additional FLT3 PROTACs. Their development requires knowledge on how they affect primary human leukemic cells with mutant FLT3 and if normal human immune cells are sensitive to such agents.

Numerous PROTACs are evaluated as drug candidates for blood malignancies [26]. Since current data suggest that tumor cells can develop resistance to PROTACs, multiple TPDs seem necessary to develop their full therapeutic potential [27]. The reported TPDs against hyperactive FLT3 is still limited to PROTACs. Moreover, current TPDs do not discriminate between the various cellular forms of FLT3-ITD. FLT3-ITD occurs at the endoplasmic reticulum (ER) as a hyper-phosphorylated, hypo-glycosylated form and when inhibited as membrane-bound, hypo-phosphorylated, hyper-glycosylated form (equals the localization of wild-type FLT3) [1, 2]. PROTACs which specifically target ER-located FLT3-ITD, the most oncogenic mutant of FLT3, have not been discovered. There is also little knowledge about cellular parameters that determine the effectiveness of TPDs beyond the induction of proximity of FLT3 to the ubiquitin-proteasome-system. Identifying such pathways may allow an optimal therapeutic usage of FLT3 PROTACs.

We report nanomolar FLT3-ITD isoform-specific TPDs that are based on the rationally designed FLT3 inhibitor MA68 and preferentially eliminate ER-bound FLT3-ITD. MA49 is a VHL-based PROTAC and MA50 is the first reported HyT for FLT3. Nanomolar doses of MA49 and MA50 induce apoptosis of leukemic cell lines and primary human AML cells with FLT3-ITD, with MA49 being two- to threefold more effective. These agents do not affect c-KIT-dependent blood cell proliferation, primary murine hematopoietic stem/progenitor cells, and the differentiation of normal human immune cells. An inactive stereoisomer of MA49 that cannot recruit VHL and genetic elimination of VHL confirm the molecular selectivity and mode of action of MA49. Mechanistically, MA49- and MA50-induced apoptosis and FLT3-ITD degradation are linked to a stabilization of the pro-apoptotic BCL2-family protein BIM. This results in the depletion of molecular chaperones of the heat shock protein (HSP) family. In an in vivo experiment, MA49 halted leukemia cell proliferation.

## Methods

The full description of all materials and methods can be found in the accompanying Supplementary text file “Materials and Methods”. Additional details can be found in references that are cited therein [28–31].

## Results

### Identification of specific degraders of FLT3-ITD

**Fig. 1:**
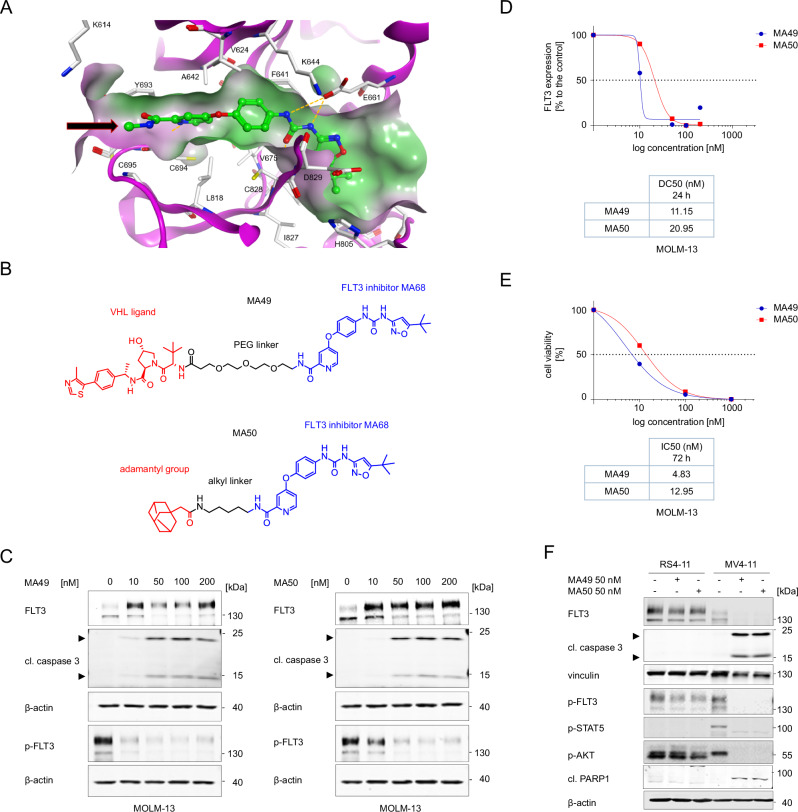
Biological characterization of the FLT3 TPDs MA49 and MA50. **A** Interaction of MA68 (green colored carbon atoms) at the ATP binding pocket of FLT3 (PDB ID 4RT7). Docking was carried out as described [54]. Hydrogen bonds are shown as orange dashed lines. The molecular surface of the binding pocket is colored according to hydrophobicity (polar regions colored magenta, hydrophobic regions colored green). The orange arrow indicates the exit vector for designing MA68-based PROTACs. **B** Structures of MA49 and MA50, including the FLT3 inhibitory scaffold from MA68, the VHL ligand in MA49, and the adamantly group mediating degradation-prone protein aggregation by MA50. **C** MOLM-13 cells were incubated with 10, 50, 100, and 200 nM MA49 or MA50 for 24 h (0, untreated control sample). Lysates of these cells were subjected to immunoblot analyses for FLT3, pY591-FLT3, and cleaved caspase-3. The protein levels of β-actin were determined to verify equal loading of samples on all tested membranes. The data are representative for the outcome of three independent experiments; cl., cleaved form, arrows point to the cleavage fragments of active caspase-3; p-, phosphorylated; kDa, molecular weight in kilodalton. **D** DC_50_ values for FLT3 degradation by MA49 and MA50 in MOLM-13 cells were determined by quantitative immunoblot using the Odyssey system. The cells were treated as stated in **B**) and lysates were analyzed for FLT3-ITD and β-actin. The values for FLT3 were normalized to β-actin and the DC_50_ values were calculated with GraphPad Prism 6. **E** IC_50_ values for apoptosis induction by MA49 and MA50 in MOLM-13 cells were determined by annexin-V and PI staining and flow cytometry. The cells were treated with 10, 100, or 1000 nM MA49 or MA50 for 72 h. IC_50_ values were calculated with GraphPad Prism 6. **F** RS4-11, MV4-11, and MOLM-13 cells were treated with 50 nM MA49 or MA50 for 24 h (+, treated; -, untreated). Lysates of these cells were subjected to immunoblot analyses for FLT3, cleaved caspase-3 (activated form upon cleavage of its autoinhibitory domain), cleaved PARP1, pY591-FLT3, pY694-STAT5, and pS473-AKT. The protein levels of vinculin and β-actin were determined to verify equal loading of samples on all tested membranes. The data are representative for the outcome of two independent experiments; cl., cleaved; p-, phosphorylated; kDa, molecular weight in kilodalton, arrows point to the cleavage fragments of active caspase-3.

By merging the pharmacophores of sorafenib and quizartinib, we synthesized the novel FLT3-ITD inhibitor MA68 (Supplementary scheme 1) which binds human FLT3-ITD with a K_d_ of 12 ± 0 nM in vitro (Supplementary Fig. S1). Crystal structures of sorafenib with kinases (e.g., PDB ID 3WZE) and docking solutions for FLT3 (PDB ID 4RT7) show that the N-methyl amide part of MA68 is in the solvent-exposed region of FLT3-ITD. Thus, this part is a feasible position for introducing a linker recruiting E3 ubiquitin-ligases (Fig. 1A).

We exploited MA68 to synthesize a VHL-recruiting FLT3 PROTAC using the VHL ligand **16** (Supplementary scheme 2) and a 3-polyethylenglycole linker (Supplementary scheme 3) as well as a HyT degrader using an adamantly moiety and an n-pentyl linker group (Supplementary scheme 4). These compounds, MA49 and MA50, are active in vitro using human FLT3-ITD, but 20-fold less than MA68 (MA49, K_d_ 195 ± 75 nM, MA50 K_d_ 345 ± 115 nM; Supplementary Fig. S1). Figure 1B shows chemical structures of MA49 and MA50.

To evaluate whether MA49 and MA50 deplete FLT3-ITD, we applied 10–200 nM MA49 and MA50 to human AML cells that express FLT3-ITD and FLT3 (MOLM-13 cells). The FLT3 band that appears at 130 kDa in immunoblot analyses corresponds to the oncogenic, hypoglycosylated ER-bound FLT3-ITD. The slower migrating band at 160 kDa is the plasma membrane-located FLT3-ITD, resembling FLT3 (refs. [1, 2]). Compared to untreated cells, cells incubated with 10 nM MA49 had slightly less of the FLT3 signal at 130 kDa and a significant accumulation of the FLT3 signal at 160 kDa in immunoblot analyses (Fig. 1C). FLT3 inhibitors induce maturation of FLT3-ITD to its fully glycosylated 160 kDa isoform [1]. Treatment with 50–100 nM MA49 depleted FLT3-ITD at 130 kDa and there was less FLT3-ITD at 160 kDa than in the 10 nM treatment group. Increasing the concentration of MA49 to 200 nM attenuated both effects (Fig. 1C). This antagonism is known as hook-effect titrating away E3 ubiquitin-ligases [32]. MA50 decreased FLT3-ITD dose-dependently without a hook-effect (Fig. 1C).

We calculated the half-maximal degradation concentrations of FLT3-ITD by MA49 and MA50. The DC_50_ values are 11.2 ± 1.4 nM for MA49 and 21 ± 1.6 nM for MA50 after 24 h (Fig. 1D). Sorafenib, quizartinib, and MA68 inhibited FLT3-ITD signaling in MOLM-13 cells but were less effective in reducing the FLT3 signal at 130 kDa (Supplementary Fig. S2).

The loss of FLT3-ITD at 130 kDa was associated with a more rapidly occurring concentration-dependent loss of phosphorylated FLT3-ITD. MA49 was more effective than MA50 in reducing phosphorylated FLT3-ITD (Fig. 1C).

The reduction and inhibition of FLT3-ITD by nanomolar concentrations of MA49 and MA50 were associated with a concentration-dependent activation of the ultimate apoptosis inducer caspase-3 (Fig. 1C) and increased cell staining by the apoptosis markers annexin-V and PI (Fig. 1E). IC_50_ values for apoptosis induction were 4.8 ± 3.7 nM for MA49 and 13 ± 2.1 nM for MA50 after 72 h (Fig. 1E).

To evaluate our data in additional leukemic cell systems, we included human RS4-11 cells with wild-type FLT3 and MV4-11 cells that express FLT3-ITD homozygously. As expected, FLT3 is mainly at the plasma membrane (160 kDa form) and FLT3-ITD is predominantly at the ER (130 kDa form) (Supplementary Fig. S3A) [1, 2]. In RS4-11 cells, 50 nM MA49 and MA50 did at most weakly attenuate FLT3, phosphorylated FLT3, and its downstream signaling to AKT. In MV4-11 cells, MA49 and MA50 degraded FLT3-ITD and diminished its activating phosphorylation of STAT5 and AKT to undetectable levels. This resulted in the activation of caspase-3 by limited proteolysis. Immunoblotting for the cleavage of its substrate PARP1 verified caspase-3 activity in MV4-11 but not in RS4-11 cells (Fig. 1F).

These data show that we have generated new TPDs for FLT3-ITD that induce apoptosis in AML cells expressing this oncoprotein.

### Elimination of FLT3-ITD protein and its downstream signaling by MA49 and MA50 is superior to FLT3 inhibition

**Fig. 2:**
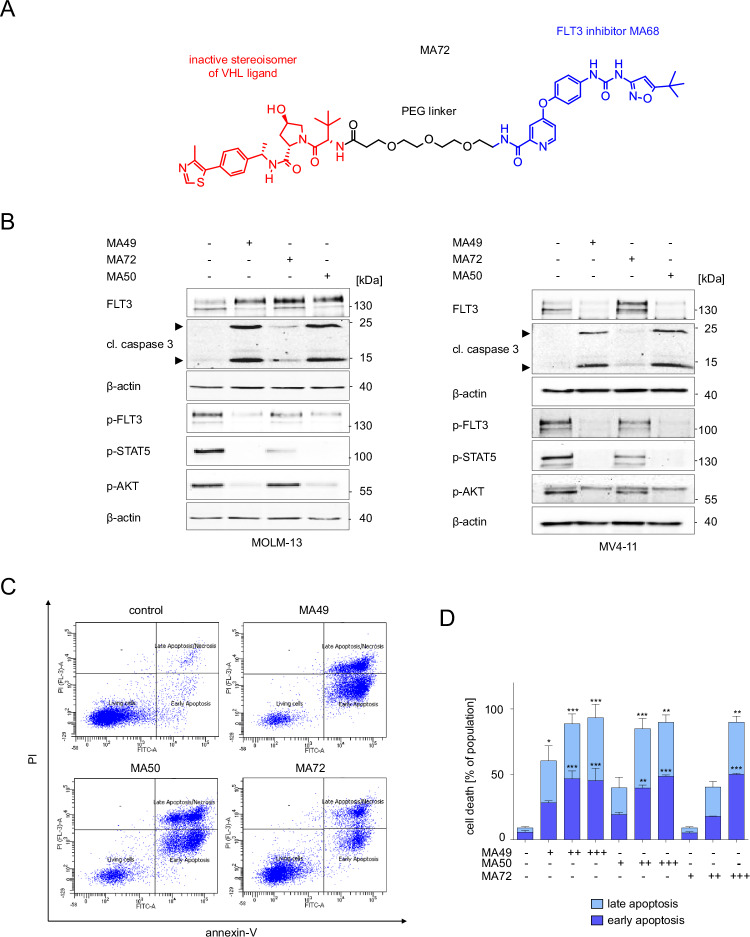
FLT3-ITD TPDs are more effective cell death inducers than corresponding sole FLT3 inhibitors. **A** Structure of MA72, which is a stereoisomer of MA49 that is unable to recruit VHL. **B** MOLM-13 and MV4-11 cells were treated with 100 nM MA49, MA72, or MA50 for 24 h (+, treated; -, untreated). Lysates of these cells were subjected to immunoblot analyses for FLT3, cleaved caspase-3, pY591-FLT3, pY694-STAT5, and pS473-AKT. The protein levels of β-actin were determined to verify equal loading of samples on all tested membranes. The data are representative for the outcome of three independent experiments; cl., cleaved form, arrows point to the cleavage fragments of active caspase-3; p-, phosphorylated; kDa, molecular weight in kilodalton. **C** The panel shows original flow cytometry data. MOLM-13 cells were treated with 100 nM MA49, MA50, or MA72 for 72 h and analyzed for apoptosis by annexin-V and PI staining using flow cytometry. **D** Apoptosis analysis of MOLM-13 cells by annexin-V and PI staining with flow cytometry. Treatments were 10, 100, and 1000 nM MA49, MA50, and MA72 for 72 h. The data are representative for the outcome of three independent experiments; -, untreated, +, 10 nM, ++ 100 nM, +++ 1000 nM. Two-way ANOVA statistical test was used to determine statistical significance; *p < 0.05; **p < 0.01; ***p < 0.001; ****p < 0.0001.

Compounds with an inactive stereoisomer of the VHL ligand are appropriate negative controls for VHL-based PROTACs. We synthesized such a negative control molecule for MA49 and called it MA72 (Fig. 2A). We incubated MOLM-13 and MV4-11 cells with 100 nM MA49, MA72, and MA50. MA72 inhibited the activating phosphorylation of FLT3-ITD and its downstream signaling to STAT5 and AKT and promoted the accumulation of FLT3-ITD at 160 kDa (Fig. 2B). These data verify the on-target activity of its FLT3 inhibitor part. Unlike MA49, MA72 did not reduce the FLT3 signal at 130 kDa in MOLM-13 cells and even augmented it in MV4-11 cells (Fig. 2B). These data verify that the elimination of FLT3-ITD by MA49 depends on the intact VHL ligand, i.e., the PROTAC nature of MA49.

Concerning FLT3-ITD signaling, MA49 and MA50 were more potent inhibitors of the p-FLT3-p-STAT5/p-AKT signaling cascades than MA72. These differences were most evident for p-AKT (Fig. 2B).

Next, we compared how MA49, MA50, and MA72 affected leukemic cell survival. Unlike MA72, MA49 and MA50 induced cleavage of caspase-3 markedly after 24 h in MV4-11 and MOLM-13 cells (Fig. 2B). Annexin-V/PI staining corroborated that the FLT3 degraders MA49 and MA50 were stronger apoptosis inducers than MA72 (Fig. 2C). Compared to MA49 (IC_50_ 4.83 nM) and MA50 (IC_50_ 12.95 nM), about 10-to-20-fold higher doses of MA72 (IC_50_ 118.1 nM) were necessary to induce apoptosis in MOLM-13 cells after 72 h (Fig. 2D).

To corroborate that MA49 triggers a VHL-based loss of FLT3-ITD, we carried out a genetic loss-of-function experiment. We depleted VHL in MV4-11 cells by siRNAs and treated the cells with MA49 and MA72. We found that the MA49-mediated degradation of FLT3-ITD was rescued when VHL was eliminated. During these experiments, we additionally noted that VHL was consumed in MV4-11 cells upon treatment with MA49 (Supplementary Fig. S3B).

These data show that MA49 is a VHL-based FLT3 PROTAC. The loss of FLT3-ITD signaling appears responsible for the superior pro-apoptotic effects of MA49 and MA50 when compared to MA72.

### MA49 and MA50 selectively kills cultured cells with FLT3-ITD

**Fig. 3:**
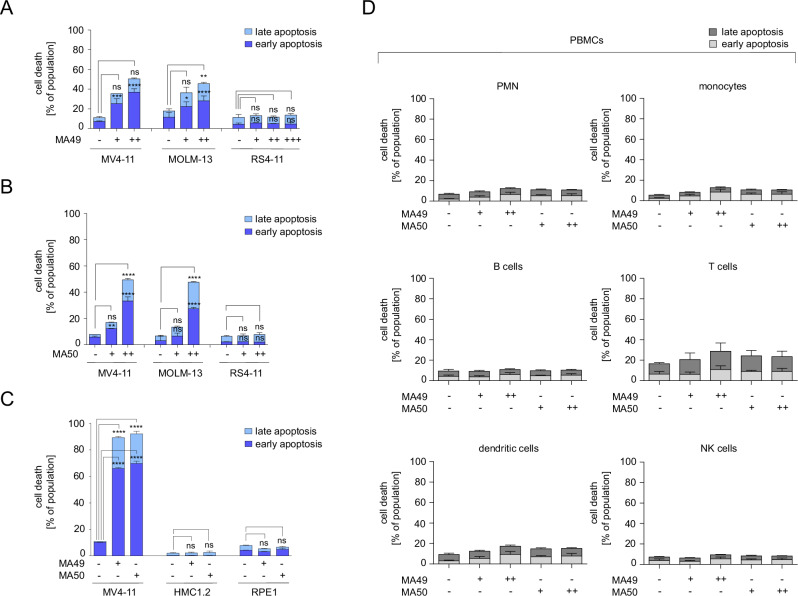
MA49 and MA50 are specifically toxic for AML cells carrying FLT3-ITD. **A** MV4-11, MOLM-13, and RS4-11 cells were treated with 10, 100, or 1000 nM MA49 for 24 h and analyzed for apoptosis by annexin-V and PI staining using flow cytometry. The data are representative for the outcome of two independent experiments; +, 10 nM, ++ 100 nM, +++ 1000 nM. **B** MV4-11, MOLM-13, and RS4-11 cells were treated with 10 or 100 nM MA50 for 24 h and analyzed for apoptosis by annexin-V and PI staining using flow cytometry. The data are representative for the outcome of two independent experiments; +, 10 nM, ++ 100 nM. **C** MV4-11, HMC1.2 (carrying c-KIT with the activating mutations G560V and D816V), and RPE1 cells were treated with 50 nM MA49 or MA50 for 72 h and analyzed for apoptosis by annexin-V and PI staining using flow cytometry. The data are representative for the outcome of two independent experiments. **D** Apoptosis analysis of PBMC subpopulations after treatment with 20 or 50 nM of MA49 or MA50 for 24 h; PBMC, peripheral blood mononuclear cells; PMN, polymorphonuclear leukocytes; NK cells, natural killer cells; -, untreated, +, 20 nM, ++ 50 nM. The data are representative for the outcome of four independent experiments. Two-way ANOVA statistical test was used to determine statistical significance; ns, not significant; *p < 0.05; **p < 0.01; ***p < 0.001; ****p < 0.0001.

To extend these observations, we analyzed the impact of MA49 and MA50 on the survival of leukemic cell lines dependent on their FLT3 status. Both compounds killed MV4-11 and MOLM-13 cells, but spared RS4-11 cells (Fig. 3A, B). These results are consistent with data shown in Figs. 1C–F, 2B–D.

To evaluate the specificity of MA49 and MA50 further, we used human mast cells (HMC1.2) and human retinal pigment epithelial cells (RPE1; immortalized by telomerase). HMC1.2 cells grow dependent on activated c-KIT. MA49 and MA50 did not affect the survival and proliferation of such cells, confirming that these TPDs selectively affect leukemic cells with FLT3-ITD (Fig. 3C, Supplementary Fig. S3C). Immunoblotting showed that MA49 and MA50 did not affect total and phosphorylated c-KIT (Supplementary Fig. S3D).

To determine the impact of MA49 and MA50 on normal blood cells, we applied them to human peripheral blood mononuclear cells (PBMC) from healthy donors. Considering that PBMCs comprise several immune cell types, [33] we analyzed polymorphonuclear leukocytes (PMNs), monocytes, B cells, T cells, dendritic cells, and natural killer (NK) cells. MA49 and MA50 did not induce apoptosis in these cell populations after 24 h (Fig. 3D) and 48 h (Supplementary Fig. S3E).

These findings illustrate that MA49 and MA50 are isoform-specific FLT3-ITD degraders that specifically kill leukemic cells with mutant FLT3.

### MA49 and MA50 downregulate molecular chaperones and ER stress proteins

**Fig. 4:**
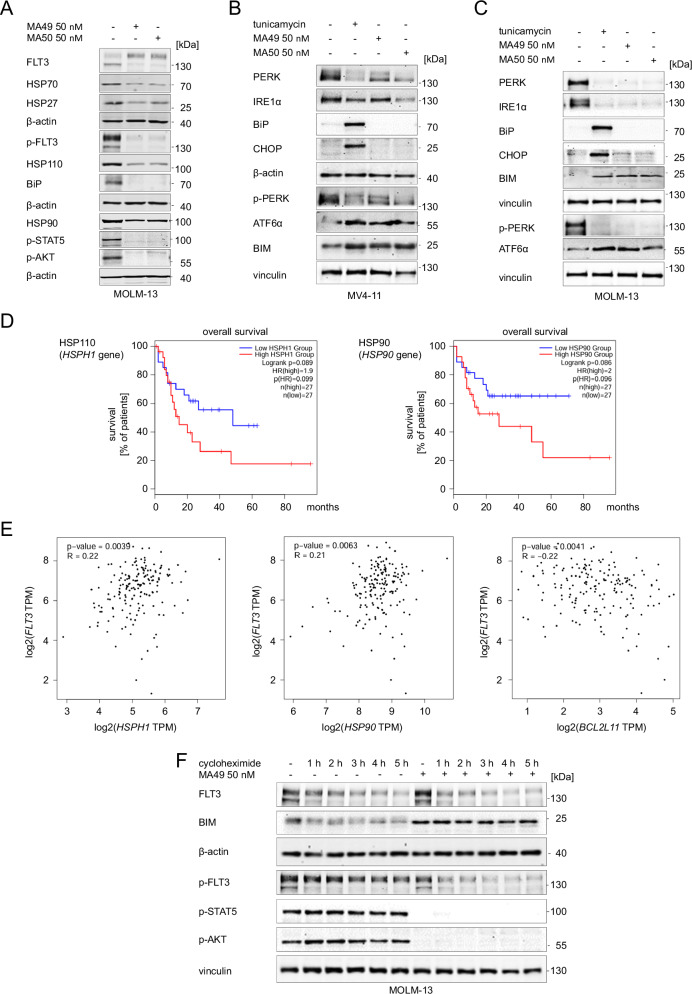
MA49 and MA50 regulate heat shock- and ER-stress-related proteins in AML cells with FLT3-ITD. **A** MOLM-13 cells were treated with 50 nM MA49 or MA50 for 24 h and analyzed by immunoblot for the expression of FLT3, HSP110, HSP90, HSP70, HSP27, BiP, pY591-FLT3, pY694-STAT5, and pS473-AKT. The protein levels of β-actin were determined to verify the equal loading of samples. The data are representative for the outcome of three independent experiments. **B**, **C** Immunoblot shows the expression of PERK, IRE1α, BiP, CHOP, pT982-PERK, BIM, and ATF6α (activated form upon cleavage in Golgi during ER stress) after treatment with 0.5 µg/ml tunicamycin or 50 nM MA49 or MA50 for 24 h in (**B**) MV4-11 or (**C**) MOLM-13 cells. The protein levels of β-actin or vinculin were determined to verify equal loading of samples. The data are representative for the outcome of two independent experiments; +, treated; -, untreated; p-, phosphorylated; kDa, molecular weight in kilodalton. **D** The GEPIA2 database was analyzed for a correlation between overall survival of AML patients and the levels of HSPs and ER stress proteins. We found significant associations of HSP110 (encoded by the *HSPH1* gene) and HSP90 (encoded by the *HSP90* gene) expression levels in leukemia cells and patient survival. The analysis included 54 patients in total, with 50% in the high and 50% in the low expressing groups (*p* = 0.089–0.086). **E** This database contains data showing an association of *FLT3* gene expression and mRNA transcripts encoding HSP110, HSP90, and BIM (*p* = 0.0039–0.0063). Positive R values indicate positive coregulation of gene expression; negative R values indicate negative coregulation of gene expression; TPM, transcripts per million reads. The graphs show the Pearson correlation coefficients. Such correlation coefficients describe linear correlations between two sets of data. GEPIA2 uses the non-log scale for calculation and use the log-scale axis for visualization. **F** MOLM-13 cells were treated with 10 μg/ml cycloheximide +/− 50 nM MA49 for 1–5 h. Lysates were analyzed by immunoblot for the expression of FLT3, BIM, pY591-FLT3, pY694-STAT5, and pS473-AKT. The protein levels of β-actin or vinculin were determined to verify equal loading of samples; +, treated; -, untreated; p-, phosphorylated; kDa, molecular weight in kilodalton.

The cellular heat shock response (HSR) is activated upon cellular stress to maintain protein homeostasis. A main sensor of the HSR, the molecular chaperone heat shock protein 90 kDa (HSP90) is frequently overexpressed in AML and stabilizes the ER-located FLT3-ITD [34, 35]. We hypothesized that MA49 and MA50 modulated the HSR. To test this, we treated MV4-11 and MOLM-13 cells with MA49 or MA50 for 24 h and analyzed the expression of molecular chaperones. MA49 and MA50 downregulated HSP70, HSP27, HSP110, BIP, and HSP90 (Fig. 4A). MA72 had a weak effect in MOLM-13 cells (Supplementary Fig. S4A) or no effect in MV4-11 cells (Supplementary Fig. S4B) on these HSPs.

We next assessed if the reduction of the ER-bound FLT3-ITD triggered an ER stress response. We investigated the pro-apoptotic ER stress response markers PKR-like endoplasmic reticulum kinase (PERK), inositol requiring enzyme 1α/β (IRE1α), activating transcription factor 6 (ATF6), the C/EBP homologous protein transcription factor (CHOP), the pro-apoptotic BH3-only protein BIM, and the ER-localized molecular chaperone immunoglobulin heavy chain binding protein (BiP). This ER-localized HSP70 paralogue acts as primary ER stress sensor [36, 37]. We neither detected increased levels or phosphorylation of the ER stress sensors PERK and IRE1α in MV4-11 (Fig. 4B) and MOLM-13 cells (Fig. 4C), nor upregulation of the major ER stress target CHOP. However, MA49 and MA50 induced cleavage of ATF6 to its active 50 kDa fragment, which regulates cellular ER stress responses [38]. The cleavage of ATF6 was induced comparably by FLT3-ITD degraders and the ER-stress inducer tunicamycin. Tunicamycin induced BiP, but MA49 and MA50 reduced BiP in MV4-11 (Fig. 4B) and MOLM-13 cells (Fig. 4C). In both cell types, tunicamycin, MA49, and MA50 induced BIM (Fig. 4B, C).

We asked if the levels of these chaperones and ER proteins are associated with AML patient survival and whether there is a link between FLT3 and BIM. Analyzing RNA-sequencing data for 54 AML patients in the GEPIA2 database, we noted that high mRNA expression levels of *HSP110* and *HSP90* are associated with a significantly reduced overall survival of AML patients (Fig. 4D; *p* < 0.1). Using this database, we further found that *FLT3* expression is positively linked to *HSP90* and *HSP110* expression and negatively correlated with the expression of *BCL2L11* (encoding BIM) (Fig. 4E; *p* < 0.01, Supplementary Fig. S4C). Similar Pearson (Fig. 4E) and Spearman correlation coefficient values (Supplementary Fig. S4C) indicate linear relationships between *FLT3* and *HSP90*/*HSP110*/*BCL2L11*.

These results illustrate that MA49 and MA50 reduce molecular chaperones, modulate ER stress pathways, and augment pro-apoptotic BIM levels.

### MA49 and MA50 induce FLT3-ITD degradation dependent on BIM and HSP90 activity

BIM is a key apoptosis initiator in FLT3-ITD-positive cells that are treated with FLT3 inhibitors [39]. These reports and our observation that MA49 and MA50, but not MA72, upregulated BIM in MV4-11 and MOLM-13 cells (Fig. 4B, C; Supplementary Fig. S4A, B) made us speculate that BIM plays a role in MA49- and MA50-mediated apoptosis. We analyzed whether BIM accumulation was a cause of its transcriptional induction or protein stabilization. We treated MOLM-13 cells with the de novo protein synthesis inhibitor cycloheximide for 1–5 h and MA49. Cycloheximide did not affect the induction of BIM by MA49 (Fig. 4F). This result suggests that MA49 stabilizes BIM on a post-translational level rather than by transcriptional upregulation.

**Fig. 5:**
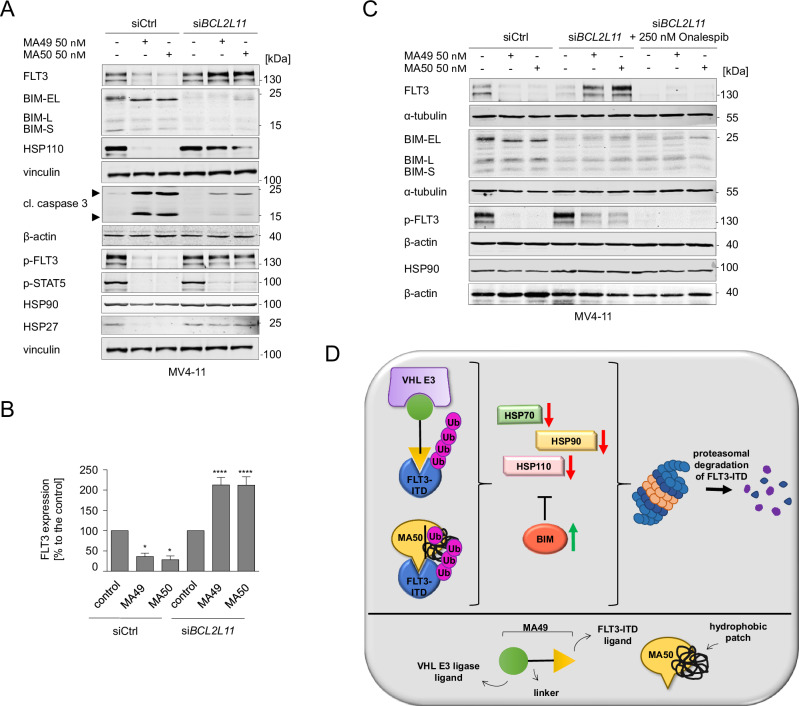
BIM regulates MA49- and MA50-mediated FLT3-ITD degradation and heat shock protein levels. **A** MV4-11 cells were transfected with siRNA against the *BCL2L11* mRNA to knock down the BIM protein. Cells without and with BIM knockdown were treated with 50 nM MA49 or MA50 for 24 h and analyzed by immunoblot for FLT3, BIM, pY591-FLT3, pY694-STAT5, cleaved (cl.) caspase-3, HSP110, HSP90, and HSP27. The protein levels of vinculin and β-actin were determined to verify the equal loading of samples. The data are representative for the outcome of four independent experiments; +, treated; -, untreated; cl., cleaved; p-, phosphorylated; kDa, molecular weight in kilodalton; siCtrl, siRNA control; si*BCL2L11*, siRNA targeting BIM, arrows point to the cleavage fragments of active caspase-3. **B** Quantification of FLT3 expression from **A**; siCtrl, siRNA control; si*BCL2L11*, BIM knockdown. One-way ANOVA statistical test was used to determine statistical significance; *p < 0.05; **p < 0.01; ***p < 0.001; ****p < 0.0001. **C** MV4-11 cells transfected with siRNA against *siBCL2L11* +/− 1 h 250 nM Onalespib pre-treatment were treated with 50 nM MA49 or MA50 for 24 h and analyzed by immunoblot for FLT3, BIM, pY591-FLT3, and HSP90 expression; siCtrl, siRNA control; si*BCL2L11*, BIM knockdown; kDa, molecular weight in kilodalton. The protein levels of α-tubulin and β-actin were determined to verify the equal loading of samples. The data are representative for the outcome of two independent experiments; +, treated; -, untreated; cl., cleaved; p-, phosphorylated; kDa, molecular weight in kilodalton; siCtrl, siRNA control; si*BCL2L11*, siRNA targeting BIM. **D** Model showing the pharmacological mechanisms of the TPDs described in this manuscript and the associated molecular mechanisms in the upper panel; the lower panel serves as legend.

To study the role of BIM in MA49- and MA50-mediated apoptosis functionally, we knocked down BIM by transfecting siRNAs targeting the mRNA encoding BIM (si*BCL2L11*) into MV4-11 cells. This knockdown (KD) was confirmed by decreased expression of the three major BIM isoforms BIM-EL, BIM-L, and BIM-S (Fig. 5A). The MA49- and MA50-mediated downregulation of HSPs (Fig. 4A) was rescued upon KD of BIM (Fig. 5A). Additionally, MA49 and MA50 failed to degrade FLT3-ITD in cells with BIM KD (Fig. 5A, B). This was linked to an attenuated inhibition of p-FLT3-ITD and p-STAT5 by MA49 and MA50. Consistently, MA49 and MA50 less efficiently induced caspase-3 cleavage in BIM KD cells compared to control siRNA-transfected cells (Fig. 5A). MA49 and MA50 stabilized BIM-EL. This was associated with its slower migration (Fig. 5A). This indicates its reduced phosphorylation, a posttranslational modification that destabilizes BIM [40].

The persistence of FLT3-ITD in AML cells with BIM KD and the failure of the TPDs to kill them (Fig. 5A) might be driven by HSP90 that stabilizes FLT3-ITD [35]. To evaluate this hypothesis, we applied the clinically tested HSP90 inhibitor onalespib to BIM KD MV4-11 cells and treated them with MA49 or MA50 (Fig. 5C). Onalespib restored MA49/MA50-mediated FLT3-ITD degradation and the inhibition of its phosphorylation.

These results suggest a regulatory role of BIM and HSP90 in FLT3-ITD degradation and the inhibition of its downstream signaling through MA49 and MA50. Figure 5D depicts these insights as model.

### MA49 kills FLT3 mutant AML blasts and cells in vitro and in vivo without an impact on hematopoietic stem/progenitor cells

**Fig. 6:**
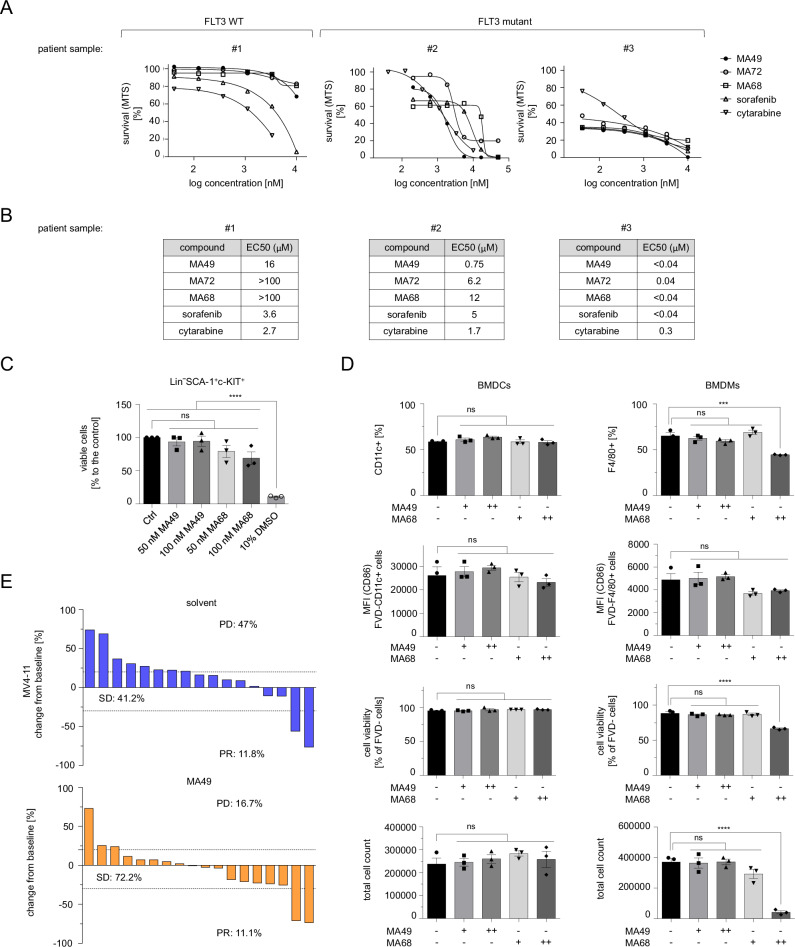
MA49 kills FLT3 mutant AML blasts and cells in vitro and in vivo without an impact on hematopoietic stem cells. **A**, **B** Serial dilutions of 50 µM top dose of MA49 versus MA72, MA68, sorafenib, and cytarabine were applied to primary ex-vivo samples from leukemia patients with wild-type FLT3 or FLT3-ITD for 72 h. Cell survival is provided as %-values compared to untreated control cells (100% survival) in the MTS-assay. **B** EC50 values for the data shown in (**A**) were determined. **C** Cell viability analysis of murine bone marrow stem cells. Bone marrow was collected from the hind legs of previously killed C57BL/6 mice. The cells were treated with the indicated concentrations of MA49, MA68, or a high concentration of 10% DMSO as a positive control for cytotoxicity for 24 h; Ctrl, untreated. One-way ANOVA statistical test was used to determine statistical significance; ns not significant; *p < 0.05; **p < 0.01; ***p < 0.001; ****p < 0.0001; *n* = 3, male and female. **D** Bone marrow cells were freshly isolated from C57BL/6 mice and cultured for 7 days in medium containing the inhibitors at 50–100 nM and supplementation with either GM-CSF to induce the differentiation of myeloid progenitor cells to BMDCs or M-CSF to induce the differentiation to BMDMs. After one week, we assessed the total cell number, viability and frequencies of CD11c+ (pan dendritic cell marker) or F4/80+ (macrophage marker) cells, the expression of their activation marker CD86, cell viability (negative for FVD), and the total cell numbers; -, untreated, +, 50 nM, ++ 100 nM; *n* = 3, male and female. **E** Waterfall plots demonstrating changes in tumor volume [%] for each individual zebrafish larvae engrafted with AML cells, from baseline (day 1 = start of the treatment) until day 3 after MV4-11 cell injection. Zebrafish larvae xenografts were treated with DMSO used as a solvent (*n* = 17 larvae; left) or 200 nM MA49 (*n* = 18 larvae; right) for 48 h; each bar reflects one individual xenograft. Numbers indicate the percentage of early larvae with progressive disease (PD), stable disease (SD) and partial response (PR) in each treatment group on day three.

The data above demonstrate that MA49 has superior activity against leukemia cells with FLT3-ITD. To evaluate the translational relevance of MA49, we assessed how MA49, MA68, sorafenib, MA72, and the clinically used chemotherapeutic cytarabine affected primary AML patient samples with FLT3 or FLT3-ITD. Of these agents, MA49 had the best anti-proliferative effect on FLT3-ITD-positive AML patient samples. Moreover, the EC_50_ values of MA49 in samples with mutant FLT3 were far below the concentrations required to eliminate AML cells with FLT3 (Fig. 6A, B).

Since MA49 is not toxic to PBMCs and their individual cell populations (Fig. 3D, Supplementary Fig. S3E), we were encouraged to determine its potential impact on primary bone marrow stem and progenitor cells. We isolated them from murine bone marrow and analyzed lineage-negative SCA-1/c-KIT-double positive cells for their viability. These hematopoietic cells correspond to CD34-positive human hematopoietic stem cells. As control, we used MA68. Compared to the positive control for cytotoxicity (10% DMSO), MA49 and MA68 did not compromise the viability of lineage-negative SCA-1/c-KIT-double positive cells. Notably, this was more evident for MA49 (Fig. 6C), indicating that it is tolerated better by hematopoietic stem and progenitor cells.

We then investigated if MA49 and MA68 affected the myelomonocytic differentiation of bone marrow-derived hematopoietic progenitors to bone marrow-derived dendritic cells (BMDCs) or bone marrow-derived macrophages (BMDMs). We assessed the total cell number, viability and frequencies of CD11c+ (pan-dendritic cell marker) or F4/80+ (macrophage marker) cells and the expression of their activation marker CD86. MA49 and MA68 did not affect the differentiation, activation, viability, and proliferation of BMDCs. This was also the case for BMDMs which received MA49. Unlike this FLT3 PROTAC, its cognate inhibitor MA68 impaired the differentiation, viability, and particularly the proliferation of macrophages significantly (Fig. 6D).

To demonstrate efficacy of MA49 in vivo, we used *Danio rerio* larvae in which we injected MV4-11 cells. Upon establishment of leukemia cell masses, the larvae were incubated with MA49 or its solvent for 48 h (Fig. 6E, Supplementary Fig. S5). Embryos were imaged before and after treatment. Embryos with at least 20% increase and 30% decrease in tumor volume were classified to have progressive disease (PD) or partial response (PR), respectively. Larvae that did not classify as PD or PR were considered to have stable disease (SD). In the control group, 41.2% of larvae had SD and 47% had PD. The response rate of xenografts upon treatment with MA49 was augmented to 72.2% SD and PD was decreased nearly threefold to 16.7% (Fig. 6E, Supplementary Fig. S5).

These data show that MA49 halts the proliferation of AML cells with FLT3-ITD, but not of normal hematopoietic cells.

## Discussion

FLT3 inhibitors are a promising therapeutic approach to treat AML [1, 2, 26]. Clinically used FLT3 inhibitors face limitations, such as a non-selective inhibition of signaling pathways beyond FLT3 and consequently dose-limiting toxic effects [1, 10, 11]. TPDs are innovative molecules that inhibit and eliminate oncoproteins in leukemic cells. This frequently results in more profound, specific, and sustained effects [26]. Nanomolar doses of MA49 and MA50 degrade FLT3-ITD and eradicate AML cells through apoptosis. The preference of these agents for the most oncogenic, ER-located FLT3-ITD may increase the overall safety and tolerability of anti-FLT3 treatment schemes.

In primary ex-vivo AML blasts, cytarabine and sorafenib did not discriminate between FLT3-ITD and FLT3. In contrast, MA49 was over 20-fold more potent against cells with FLT3-ITD than against cells with FLT3. It is likewise promising that MA49 and MA50 do not reduce FLT3 and c-KIT and have no negative impact on various immune cell types, including mast cells, dendritic cells, and NK cells. These express c-KIT [41] and NK and T cells identify and destroy malignant cells [42]. Therefore, both inhibitors hold the promise of continuous, normal hematopoiesis and immune competence. Using primary, bone marrow-derived hematopoietic stem/progenitor cells, we correspondingly demonstrate that MA49 does not affect their survival and differentiation. MA68 though impairs the survival and proliferation of such cells. This better safety profile of a FLT3 PROTAC further justifies the search and characterization of TPDs that eliminate FLT3-ITD.

Excessively high concentrations of PROTACs can saturate the ubiquitin-proteasome system and disable protein degradation efficiency [32]. This hook effect necessitates a careful titration of PROTACs in vivo. An obstacle for the clinical usage of TPDs could also be the intrinsic and acquired resistance of tumor cells due to mutations that prevent TPD-induced E3 ligase binding [27]. Hence, realizing the full clinical potential of TPDs requires the discovery of multiple degraders with different modes of actions. The HyT degrader MA50 offers several advantages for cellular testing including lower molecular weight (656 Da), and lower polar surface area ( < 150 Å) compared to PROTACs. Theoretically, this allows higher cellular permeability. However, MA49 showed higher efficacy than MA50 against FLT3-ITD, its downstream signaling, and AML cells. The VHL-based MA49 may also reduce the risk of thrombocytopenia and brain hemorrhage due to a very low expression of VHL in megakaryocytes [43]. It should additionally be mentioned that MA49 and MA50 minimize the risk of teratogenic side effects of thalidomide-derived CRBN-ligands.

HSPs stabilize oncoproteins, such as fusion proteins resulting from chromosomal translocations in leukemia, and key tumor-promoting kinases, such as FLT3, RAF, and AKT [44]. Congruently, we see that the levels of HSP90 and HSP110 inversely correlate with AML patient survival. Inhibitors of HSP70 and HSP90 are considered for leukemia therapy [45, 46], but HSP70 inhibitors have not reached the clinic because of their toxicity [47]. Most HSP90 inhibitors are also deemed clinical failures owing to toxicity and drug resistance, due to the HSP70 upregulation and epichaperomes. Common side effects of HSP90 inhibitors are liver, ocular, and gastrointestinal toxicity. HSP70 upregulation compensates the drug-increased proteotoxic stress and epichaperomes confer protein-protein networks of HSPs, co-chaperones, and client proteins [48, 49]. Both FLT3 and c-KIT are HSP90 clients, increasing the risk of hematotoxicity upon HSP90 inhibition [35, 44]. MA49 and MA50 disturb proteostasis of cells with FLT3-ITD by dysregulating HSP110, HSP90, HSP70, HSP27, and BiP, but we did not appreciate an effect of these TPDs on c-KIT.

The pro-apoptotic impact of MA49 and MA50 on AML cells with mutant FLT3 requires BIM, which controls the efflux of cytochrome c from mitochondria and the resulting activation of caspases [50]. The linkage between FLT3 and HSP90, HSP110, and BIM expression in primary AML specimen suggests that a pharmacologically amenable control of FLT3-ITD by TPDs hits general mechanisms of leukemogenesis. BIM also acts as a co-chaperone for HSP70, enhancing its anti-apoptotic activities [51]. However, MA49- and MA50-mediated BIM upregulation downregulates HSP70 and other chaperones. Since onalespib destabilizes FLT3-ITD irrespective of BIM, it will be interesting to see if HSP90 inhibitors combine favorably with MA49 and MA50 against leukemic cells.

The stabilization of BIM protein levels by MA49 and MA50 is likely caused by their negative impact on AKT signaling. AKT antagonizes BIM through its phosphorylation and subsequently accelerated proteasomal degradation [40]. As inhibition of AKT and its downstream signaling to glycogen synthase kinase-3β downregulates the expression of heat shock factor-1, the master regulator of the HSR, this might explain how MA49 and MA50 decrease HSPs [52]. The ER stress-responsive transcription factor CHOP induces BIM, but the MA49/MA50-mediated upregulation of BIM was not associated with its upregulation [53]. The accumulation of ATF6 in MA49/MA50-treated FLT3-ITD-positive cells can likewise not explain the accumulation of BIM, because ATF6 does not induce BIM [38]. It remains to be shown if the induction of BIM is linked to other ER stress signatures, such as activation of JUN-kinase or caspase-12.

MA49 and MA50 contain elements of FLT3 inhibitors and elements coupling FLT3 to the ubiquitin-proteasome-system. Knockdown of BIM prevents FLT3-ITD degradation by MA49 and MA50 and this reduces their inhibitory effect on FLT3-ITD. Thus, these compounds act as molecular degraders rather than as TKi. Given that MA68 is derived from sorafenib and quizartinib, it is likely a type II inhibitor. FLT3-ITD/TKD mutations might confer resistance to MA68 and its derivatives. We tried to obtain MV4-11 and MOLM-13 cells with acquired resistance to these compounds without success. Although this appears to be promising, further studies must be conducted to firmly rule out the development of such resistance.

In conclusion, we identified MA49 and MA50 as novel, highly efficient candidates for a potential treatment of FLT3-ITD-driven AML cells. Our studies in cultured cells, primary differentiated and undifferentiated normal hematopoietic cells, and the investigation of leukemia cell growth in an in vivo model coherently illustrate that MA49 kills leukemia cells with FLT3-ITD significantly and without a detectable negative impact on normal differentiated and undifferentiated cells and tissues. In addition, we reveal mechanistic insights into the modes of action of these new TPDs for FLT3-ITD.

## Supplementary information

REVISION_Supplementary figures_Halilovic et al

Chemical synthesis and verifications

Supplementary information File - Materials and Methods, Halilovic et al.

Immunoblots - Original Files

## Data Availability

All data are available within the main text and the Supplementary Information files. Original immunoblot files are provided, and all data are available upon scientific reasonable request.

## References

[1] Müller JP, Schmidt-Arras D. Novel approaches to target mutant FLT3 leukaemia. Cancers. 2020;12:2806. 33003568 10.3390/cancers12102806 PMC7600363

[2] Short NJ, Nguyen D, Ravandi F. Treatment of older adults with FLT3-mutated AML: emerging paradigms and the role of frontline FLT3 inhibitors. Blood Cancer J. 2023;13:142. 37696819 10.1038/s41408-023-00911-w PMC10495326

[3] Carneiro BA, El-Deiry WS. Targeting apoptosis in cancer therapy. Nat Rev Clin Oncol. 2020;17:395–417. 32203277 10.1038/s41571-020-0341-y PMC8211386

[4] Döhner H, Wei AH, Appelbaum FR, Craddock C, DiNardo CD, Dombret H, et al. Diagnosis and management of AML in adults: 2022 recommendations from an international expert panel on behalf of the ELN. Blood. 2022;140:1345–77. 35797463 10.1182/blood.2022016867

[5] Li S, Li N, Chen Y, Zheng Z, Guo Y. FLT3-TKD in the prognosis of patients with acute myeloid leukemia: a meta-analysis. Front Oncol. 2023;13:1086846. 36874106 10.3389/fonc.2023.1086846 PMC9982020

[6] Yu J, Jiang PYZ, Sun H, Zhang X, Jiang Z, Li Y, et al. Advances in targeted therapy for acute myeloid leukemia. Biomark Res. 2020;8:17. 32477567 10.1186/s40364-020-00196-2 PMC7238648

[7] Weisberg E, Meng C, Case AE, Tiv HL, Gokhale PC, Buhrlage SJ, et al. Effects of the multi-kinase inhibitor midostaurin in combination with chemotherapy in models of acute myeloid leukaemia. J Cell Mol Med. 2020;24:2968–80. 31967735 10.1111/jcmm.14927 PMC7077552

[8] Morin S, Giannotti F, Mamez AC, Pradier A, Masouridi-Levrat S, Simonetta F, et al. Real-world experience of sorafenib maintenance after allogeneic hematopoietic stem cell transplantation for FLT3-ITD AML reveals high rates of toxicity-related treatment interruption. Front Oncol. 2023;13:1095870. 37007116 10.3389/fonc.2023.1095870 PMC10050716

[9] Roskoski R Jr. Properties of FDA-approved small molecule protein kinase inhibitors: a 2024 update. Pharm Res. 2024;200:107059. 10.1016/j.phrs.2024.107059 38216005

[10] Abdel-Aziz AK, Dokla EME, Saadeldin MK. FLT3 inhibitors and novel therapeutic strategies to reverse AML resistance: an updated comprehensive review. Crit Rev Oncol Hematol. 2023;191:104139. 37717880 10.1016/j.critrevonc.2023.104139

[11] Erba HP, Montesinos P, Kim HJ, Patkowska E, Vrhovac R, Zak P, et al. Quizartinib plus chemotherapy in newly diagnosed patients with FLT3-internal-tandem-duplication-positive acute myeloid leukaemia (QuANTUM-First): a randomised, double-blind, placebo-controlled, phase 3 trial. Lancet. 2023;401:1571–83. 37116523 10.1016/S0140-6736(23)00464-6

[12] Wang X, DeFilippis RA, Yan W, Shah NP, Li HY. Overcoming secondary mutations of type II kinase inhibitors. J Med Chem. 2024;67:9776–88. 38837951 10.1021/acs.jmedchem.3c01629 PMC11586107

[13] Weisberg E, Meng C, Case AE, Sattler M, Tiv HL, Gokhale PC, et al. Comparison of effects of midostaurin, crenolanib, quizartinib, gilteritinib, sorafenib and BLU-285 on oncogenic mutants of KIT, CBL and FLT3 in haematological malignancies. Br J Haematol. 2019;187:488–501. 31309543 10.1111/bjh.16092 PMC7887860

[14] Levis M, Perl AE. Gilteritinib: potent targeting of FLT3 mutations in AML. Blood Adv. 2020;4:1178–91. 32208491 10.1182/bloodadvances.2019000174 PMC7094008

[15] Dong G, Ding Y, He S, Sheng C. Molecular glues for targeted protein degradation: from serendipity to rational discovery. J Med Chem. 2021;64:10606–20. 34319094 10.1021/acs.jmedchem.1c00895

[16] Hanzl A, Casement R, Imrichova H, Hughes SJ, Barone E, Testa A, et al. Functional E3 ligase hotspots and resistance mechanisms to small-molecule degraders. Nat Chem Biol. 2023;19:323–33. 36329119 10.1038/s41589-022-01177-2 PMC7614256

[17] Pettersson M, Crews CM. PROteolysis TArgeting Chimeras (PROTACs) - Past, present and future. Drug Discov Today Technol. 2019;31:15–27. 31200855 10.1016/j.ddtec.2019.01.002 PMC6578591

[18] Luh LM, Scheib U, Juenemann K, Wortmann L, Brands M, Cromm PM. Prey for the proteasome: targeted protein degradation—a medicinal chemist’s perspective. Angew Chem Int Ed Engl. 2020;59:15448–66. 32428344 10.1002/anie.202004310 PMC7496094

[19] Burslem GM, Song J, Chen X, Hines J, Crews CM. Enhancing antiproliferative activity and selectivity of a FLT-3 inhibitor by proteolysis targeting chimera conversion. J Am Chem Soc. 2018;140:16428–32. 30427680 10.1021/jacs.8b10320

[20] Zhai J, Li C, Sun B, Wang S, Cui Y, Gao Q, et al. Sunitinib-based proteolysis targeting chimeras (PROTACs) reduced the protein levels of FLT-3 and c-KIT in leukemia cell lines. Bioorg Med Chem Lett. 2022;78:129041. 36332882 10.1016/j.bmcl.2022.129041

[21] Cao S, Ma L, Liu Y, Wei M, Yao Y, Li C, et al. Proteolysis-targeting chimera (PROTAC) modification of Dovitinib enhances the antiproliferative effect against FLT3-ITD-positive acute myeloid leukemia cells. J Med Chem. 2021;64:16497–511. 34694800 10.1021/acs.jmedchem.1c00996

[22] Chen Y, Yuan X, Tang M, Shi M, Yang T, Liu K, et al. Degrading FLT3-ITD protein by proteolysis targeting chimera (PROTAC). Bioorg Chem. 2022;119:105508. 34959180 10.1016/j.bioorg.2021.105508

[23] Ohoka N, Suzuki M, Uchida T, Tsuji G, Tsukumo Y, Yoshida M, et al. Development of Gilteritinib-based chimeric small molecules that potently induce degradation of FLT3-ITD protein. ACS Med Chem Lett. 2022;13:1885–91. 36518702 10.1021/acsmedchemlett.2c00402 PMC9743425

[24] Liu W, Bai Y, Zhou L, Jin J, Zhang M, Wang Y, et al. Discovery of LWY713 as a potent and selective FLT3 PROTAC degrader with in vivo activity against acute myeloid leukemia. Eur J Med Chem. 2024;264:115974. 38007910 10.1016/j.ejmech.2023.115974

[25] Reznickova E, Krajcovicova S, Perina M, Kovalova M, Soural M, Krystof V. Modulation of FLT3-ITD and CDK9 in acute myeloid leukaemia cells by novel proteolysis targeting chimera (PROTAC). Eur J Med Chem. 2022;243:114792. 36191408 10.1016/j.ejmech.2022.114792

[26] Casan JML, Seymour JF. Degraders upgraded: the rise of PROTACs in hematological malignancies. Blood. 2024;143:1218–30. 10.1182/blood.2023022993 38170175

[27] Schröder M, Renatus M, Liang X, Meili F, Zoller T, Ferrand S, et al. DCAF1-based PROTACs with activity against clinically validated targets overcoming intrinsic- and acquired-degrader resistance. Nat Commun. 2024;15:275. 38177131 10.1038/s41467-023-44237-4 PMC10766610

[28] Zeyn Y, Hausmann K, Halilovic M, Beyer M, Ibrahim HS, Brenner W, et al. Histone deacetylase inhibitors modulate hormesis in leukemic cells with mutant FMS-like tyrosine kinase-3. Leukemia. 2023;37:2319–23. 37735559 10.1038/s41375-023-02036-2 PMC10624624

[29] Hieber C, Mustafa AM, Neuroth S, Henninger S, Wollscheid HP, Zabkiewicz J, et al. Inhibitors of the tyrosine kinases FMS-like tyrosine kinase-3 and WEE1 induce apoptosis and DNA damage synergistically in acute myeloid leukemia cells. Biomed Pharmacother. 2024;177:117076. 38971011 10.1016/j.biopha.2024.117076

[30] Seiboldt T, Zeiser C, Nguyen D, Celikyurekli S, Herter S, Najafi S, et al. Synergy of retinoic acid and BH3 mimetics in MYC(N)-driven embryonal nervous system tumors. Br J Cancer. 2024;131:763–77. 10.1038/s41416-024-02740-5 PMC11333474 38942989

[31] Tang Z, Kang B, Li C, Chen T, Zhang Z. GEPIA2: an enhanced web server for large-scale expression profiling and interactive analysis. Nucleic Acids Res. 2019;47:556–60. 10.1093/nar/gkz430 PMC6602440 31114875

[32] Moreau K, Coen M, Zhang AX, Pachl F, Castaldi MP, Dahl G, et al. Proteolysis-targeting chimeras in drug development: a safety perspective. Br J Pharm. 2020;177:1709–18. 10.1111/bph.15014 PMC7070175 32022252

[33] Kleiveland CCR Peripheral blood mononuclear cells. In: Verhoeckx K, Cotter P, Lopez-Exposito I, Kleiveland C, Lea T, Mackie A, et al., editors. The Impact of Food Bioactives on Health: in vitro and ex vivo models. Cham (CH): Springer. 2015. p. 161–7. 29787039

[34] Genest O, Wickner S, Doyle SM. Hsp90 and Hsp70 chaperones: collaborators in protein remodeling. J Biol Chem. 2019;294:2109–20. 30401745 10.1074/jbc.REV118.002806 PMC6369297

[35] Katagiri S, Chi S, Minami Y, Fukushima K, Shibayama H, Hosono N, et al. Mutated KIT tyrosine kinase as a novel molecular target in acute myeloid leukemia. Int J Mol Sci. 2022;23:4694. 35563085 10.3390/ijms23094694 PMC9103326

[36] Kopp MC, Larburu N, Durairaj V, Adams CJ, Ali MMU. UPR proteins IRE1 and PERK switch BiP from chaperone to ER stress sensor. Nat Struct Mol Biol. 2019;26:1053–62. 31695187 10.1038/s41594-019-0324-9 PMC6858872

[37] Li H, Musayev FN, Yang J, Su J, Liu Q, Wang W, et al. A novel and unique ATP hydrolysis to AMP by a human Hsp70 Binding immunoglobin protein (BiP). Protein Sci. 2022;31:797–810. 34941000 10.1002/pro.4267 PMC8927878

[38] Feral K, Jaud M, Philippe C, Di Bella D, Pyronnet S, Rouault-Pierre K, et al. ER stress and unfolded protein response in leukemia: friend, foe, or both? Biomolecules. 2021;11:199. 33573353 10.3390/biom11020199 PMC7911881

[39] Zhu R, Li L, Nguyen B, Seo J, Wu M, Seale T, et al. FLT3 tyrosine kinase inhibitors synergize with BCL-2 inhibition to eliminate FLT3/ITD acute leukemia cells through BIM activation. Signal Transduct Target Ther. 2021;6:186. 34024909 10.1038/s41392-021-00578-4 PMC8141515

[40] Manoharan S, Prajapati K, Perumal E. Natural bioactive compounds and FOXO3a in cancer therapeutics: an update. Fitoterapia. 2024;173:105807. 38168566 10.1016/j.fitote.2023.105807

[41] Stahl M, Gedrich R, Peck R, LaVallee T, Eder JP. Targeting KIT on innate immune cells to enhance the antitumor activity of checkpoint inhibitors. Immunotherapy. 2016;8:767–74. 27349976 10.2217/imt-2016-0040

[42] Gonzalez H, Hagerling C, Werb Z. Roles of the immune system in cancer: from tumor initiation to metastatic progression. Genes Dev. 2018;32:1267–84. 30275043 10.1101/gad.314617.118 PMC6169832

[43] Khan S, Zhang X, Lv DW, Zhang Q, He YH, Zhang PY, et al. A selective BCL-X PROTAC degrader achieves safe and potent antitumor activity. Nat Med. 2019;25:1938–47. 31792461 10.1038/s41591-019-0668-z PMC6898785

[44] Cabaud-Gibouin V, Durand M, Quere R, Girodon F, Garrido C, Jego G. Heat-shock proteins in leukemia and lymphoma: multitargets for innovative therapeutic approaches. Cancers. 2023;15:984. 36765939 10.3390/cancers15030984 PMC9913431

[45] Kurop MK, Huyen CM, Kelly JH, Blagg BSJ. The heat shock response and small molecule regulators. Eur J Med Chem. 2021;226:113846. 34563965 10.1016/j.ejmech.2021.113846 PMC8608735

[46] Braunstein MJ, Scott SS, Scott CM, Behrman S, Walter P, Wipf P, et al. Antimyeloma effects of the heat shock protein 70 molecular chaperone inhibitor MAL3-101. J Oncol. 2011;2011:232037. 21977030 10.1155/2011/232037 PMC3184436

[47] Mouawad N, Capasso G, Ruggeri E, Martinello L, Severin F, Visentin A, et al. Is it still possible to think about HSP70 as a therapeutic target in onco-hematological diseases? Biomolecules. 2023;13:604. 37189352 10.3390/biom13040604 PMC10135835

[48] Butler LM, Ferraldeschi R, Armstrong HK, Centenera MM, Workman P. Maximizing the therapeutic potential of HSP90 inhibitors. Mol Cancer Res. 2015;13:1445–51. 26219697 10.1158/1541-7786.MCR-15-0234 PMC4645455

[49] Chiosis G, Digwal CS, Trepel JB, Neckers L. Structural and functional complexity of HSP90 in cellular homeostasis and disease. Nat Rev Mol Cell Biol. 2023;24:797–815. 37524848 10.1038/s41580-023-00640-9 PMC10592246

[50] Parry N, Wheadon H, Copland M. The application of BH3 mimetics in myeloid leukemias. Cell Death Dis. 2021;12:222. 33637708 10.1038/s41419-021-03500-6 PMC7908010

[51] Guo ZW, Song T, Wang ZQ, Lin DH, Cao KK, Liu P, et al. The chaperone Hsp70 is a BH3 receptor activated by the pro-apoptotic Bim to stabilize anti-apoptotic clients. J Biol Chem. 2020;295:12900–9. 32651234 10.1074/jbc.RA120.013364 PMC7489912

[52] Kmiecik SW, Mayer MP. Molecular mechanisms of heat shock factor 1 regulation. Trends Biochem Sci. 2022;47:218–34. 34810080 10.1016/j.tibs.2021.10.004

[53] Hu H, Tian M, Ding C, Yu S. The C/EBP homologous protein (CHOP) transcription factor functions in endoplasmic reticulum stress-induced apoptosis and microbial infection. Front Immunol. 2018;9:3083. 30662442 10.3389/fimmu.2018.03083 PMC6328441

[54] Beyer M, Henninger SJ, Haehnel PS, Mustafa AHM, Gurdal E, Schubert B, et al. Identification of a highly efficient dual type I/II FMS-like tyrosine kinase inhibitor that disrupts the growth of leukemic cells. Cell Chem Biol. 2022;29:398–411. 34762849 10.1016/j.chembiol.2021.10.011

