# Supplementary material for: Selective degradation of mutant FMS-like tyrosine kinase-3 requires BIM-dependent depletion of heat shock proteins
Source: Leukemia. 2024 Sep 17;38(12):2561–72. doi: 10.1038/s41375-024-02405-5 (PMC11588663; doi:10.1038/s41375-024-02405-5)
Supplement: Supplementary file 1 — REVISION_Supplementary figures_Halilovic et al [file 41375_2024_2405_MOESM1_ESM.docx]

**Supplementary figures**


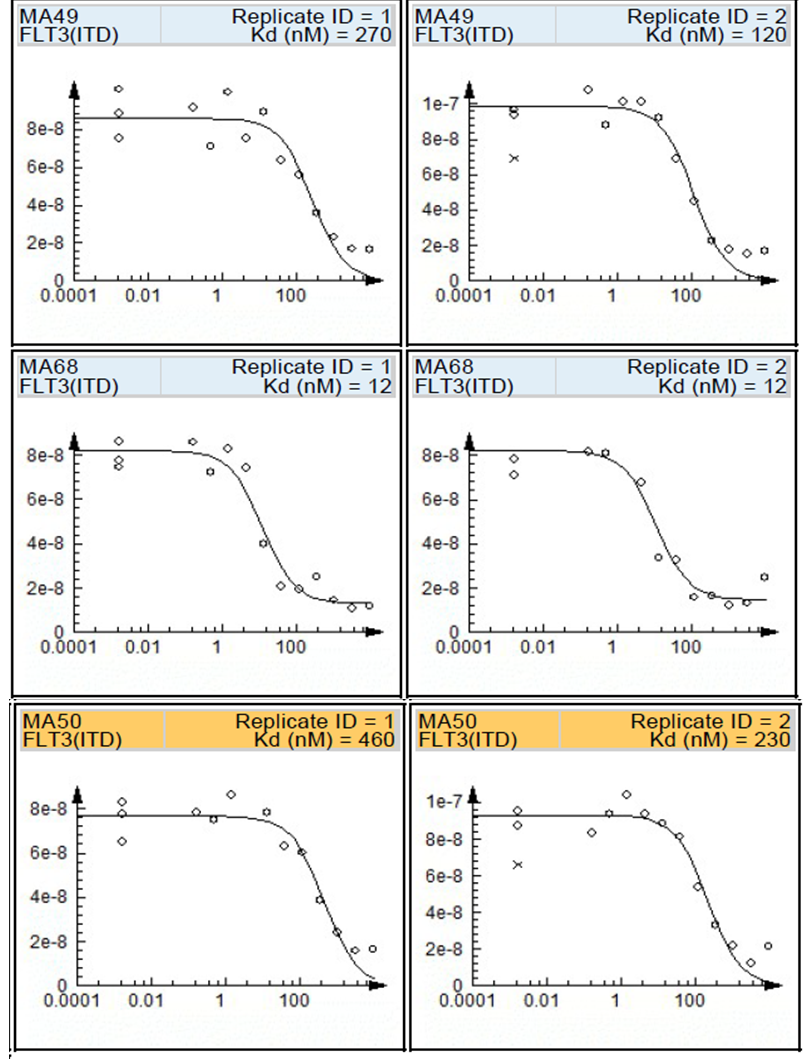


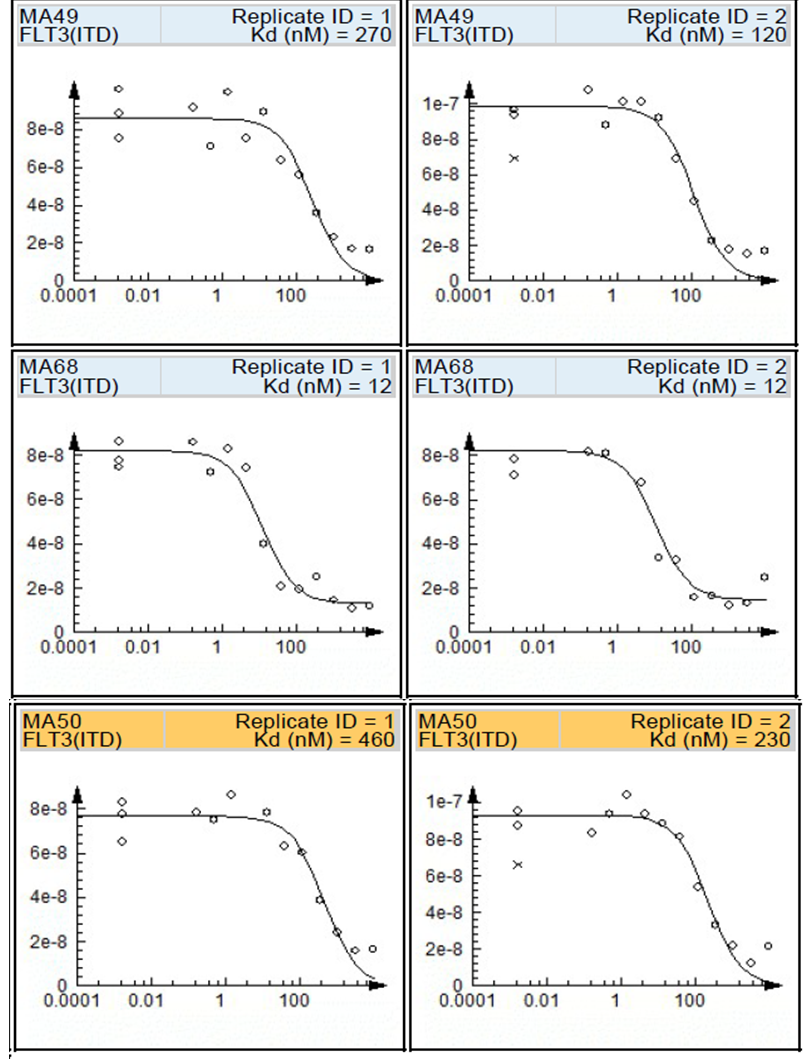


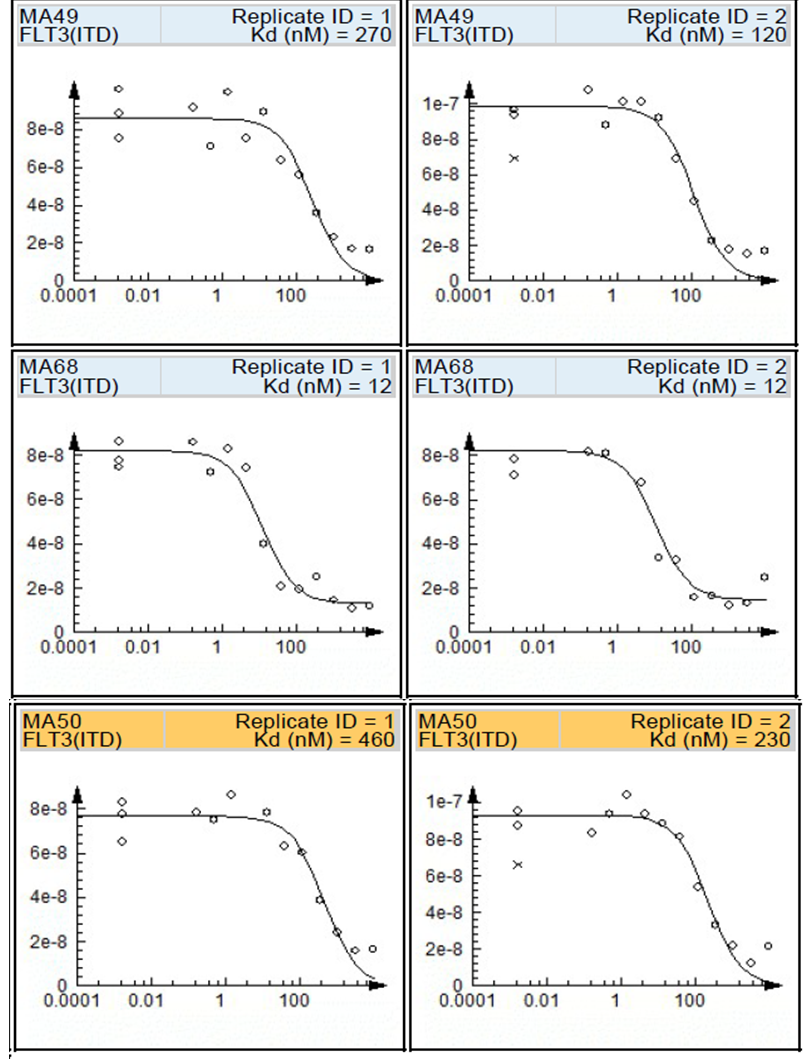


**Figure S1. Curve images for the tested kinase inhibitors.** The amount of FLT3-ITD kinase measured by qPCR (Signal; y-axis) is plotted against the corresponding compound concentration in nM in log_10_ scale (x-axis). Data points marked with an "x" were not used for Kd determination.


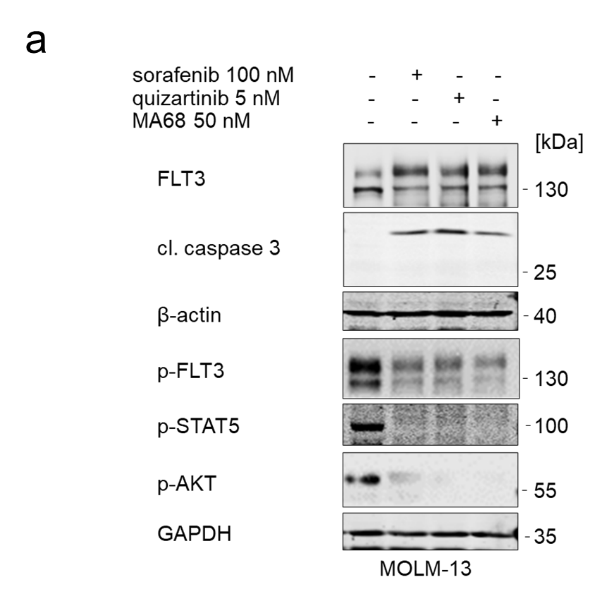


**Figure S2. Novel MA68 FLT3 inhibitor was used to synthesize MA49 and MA50 FLT3-ITD molecular degraders.** Comparison of FLT3 inhibitors sorafenib, quizartinib and MA68 in AML cells. MOLM-13 cells were treated with 100 nM sorafenib or 5 nM quizartinib or 50 nM MA68 for 24 h and analyzed by immunoblot for the expression of FLT3, cleaved caspase-3, pY591-FLT3, pY694-STAT5, and pS473-AKT. The protein levels of β-actin or GAPDH were determined to verify the equal loading of samples. The data are representative for the outcome of two independent experiments; cl., cleaved form; p-, phosphorylated; kDa, molecular weight in kilodalton.


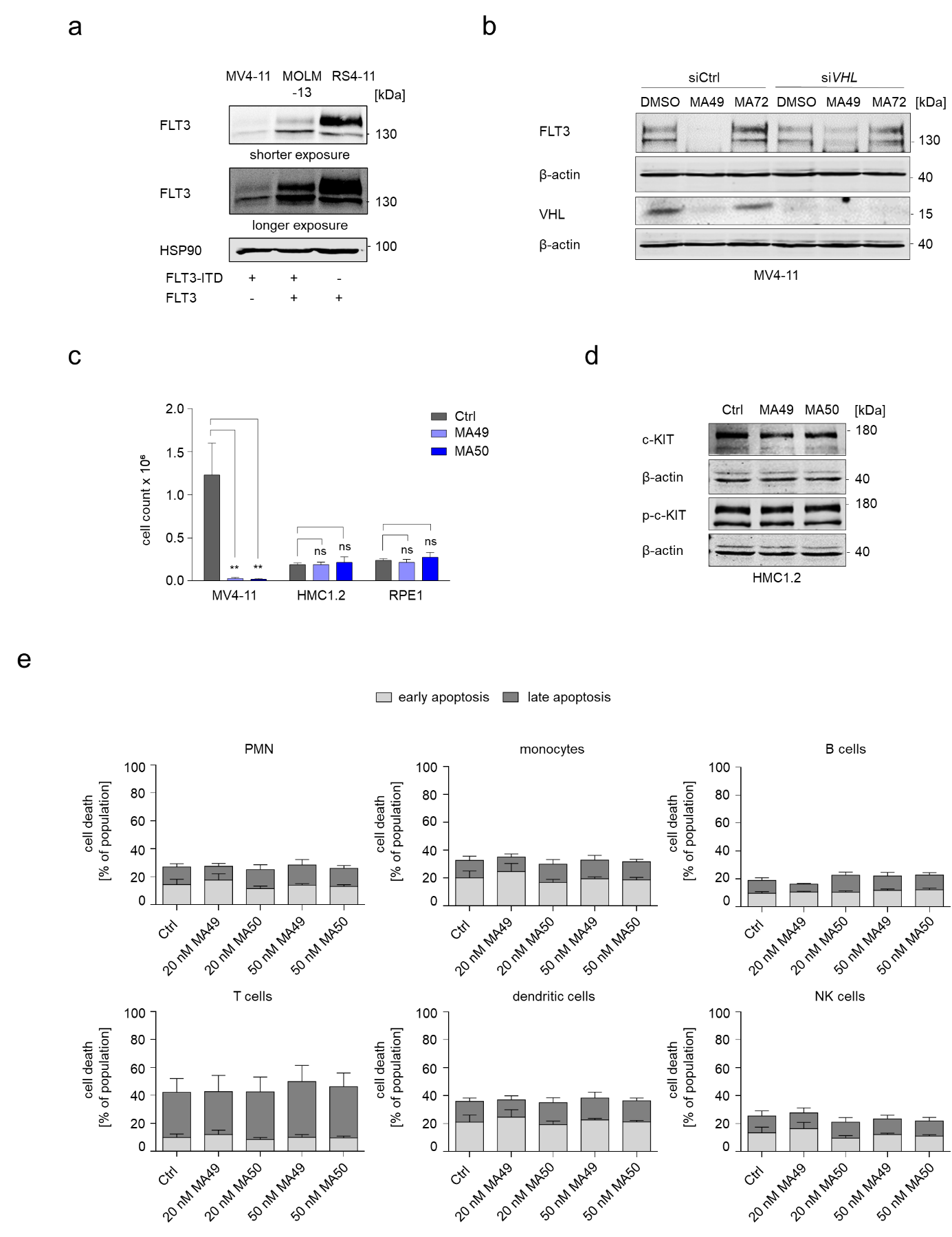


**Figure S3. MA49 and MA50 selectively target FLT3-ITD-positive cells. a** Immunoblot shows bands of FLT3-ITD and FLT3 in the leukemic cells; kDa, molecular weight in kilodalton. **b** VHL knockdown rescues MA49-mediated FLT3-ITD degradation. MV4-11 cells without or with VHL knockdown were treated with 50 nM MA49 and the negative control compound MA72 for 24 h. Lysates of the cells were analyzed by immunoblot; siCtrl, siRNA control; si*VHL*, VHL knockdown by RNAi. **c** Effects of MA49 and MA50 on the proliferation of MV4-11, HMC1.2, and RPE1 cells. Cells were treated with 50 nM MA49 and MA50 for 72 h; Ctrl, control. **d** Immunoblot shows no effect of MA49 and MA50 on the c-KIT protein and its phosphorylation levels in the HMC1.2 cell line. MA49 or MA50 were applied at 50 nM for 24 h and lysates were analyzed for the expression of c-KIT and pTyr719-c-KIT; Ctrl, control; p-, phosphorylated; kDa, molecular weight in kilodalton. **e** Apoptosis analysis of MA49- and MA50-treated PBMC subpopulations after 48 h treatment. Cells were treated with 20 or 50 nM MA49 or MA50 for 48 h; Ctrl, control; PBMC, peripheral blood mononuclear cells; PMN, polymorphonuclear leukocytes; NK cells, natural killer cells. Two-way ANOVA statistical test was used to determine significance; ns, not significant; *p, <0.05; **p, <0.01; ***p, <0.001; ****p, <0.0001.


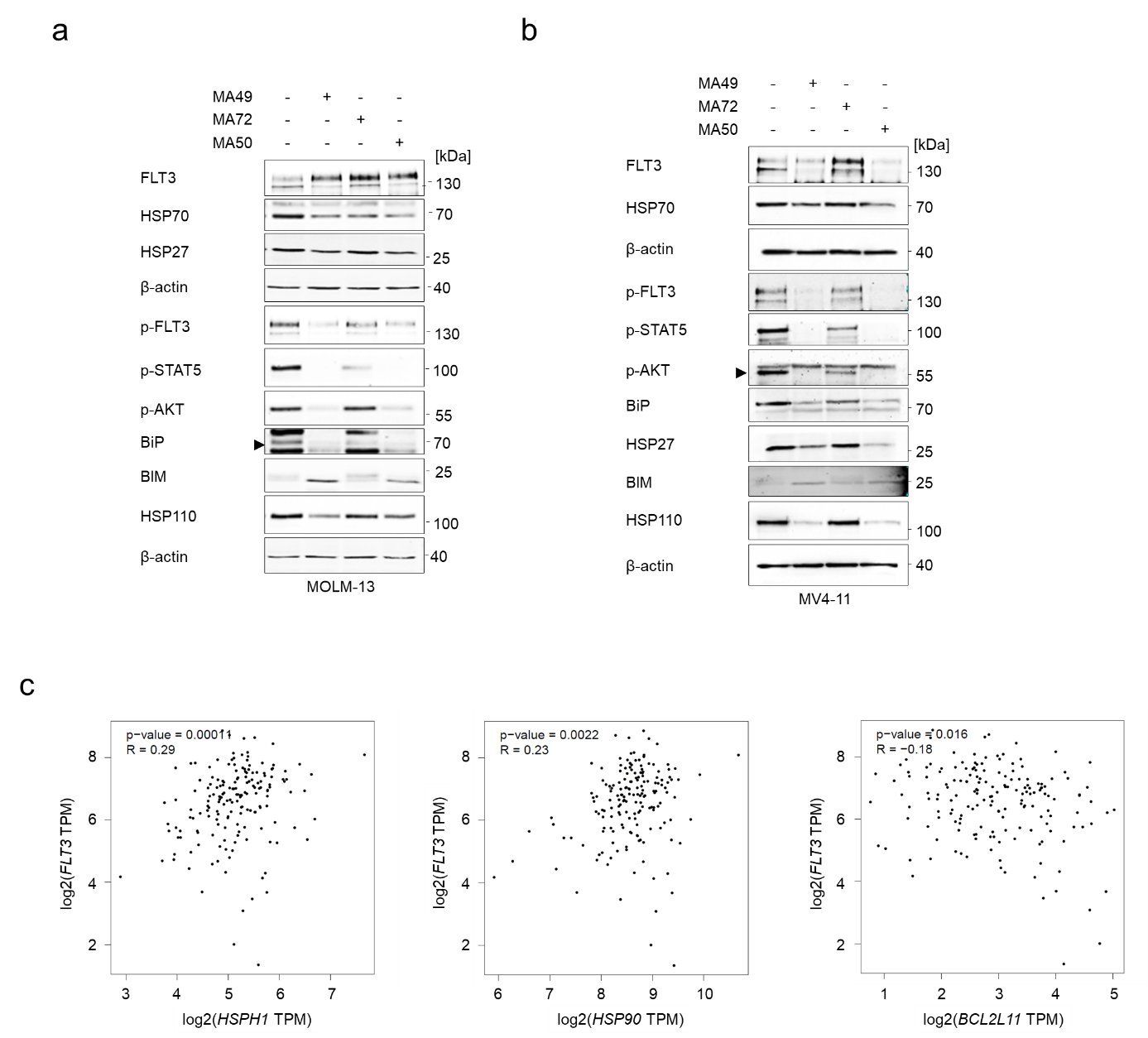


**Figure S4. MA49 and MA50 regulate heat shock response in AML cells. a, b** Immunoblot shows the expression of FLT3, HSP110, HSP70, HSP27, BiP, BIM, pY591-FLT3, pY694-STAT5, and pS473-AKT after treatment with 100 nM MA49, MA72 or MA50 for 24 h in **a** MOLM-13 and **b** MV4-11 cells. The protein levels of β-actin were determined to verify the equal loading of samples; +, treated; -, untreated; p-, phosphorylated; kDa, molecular weight in kilodalton. **c** GEPIA2 correlation analysis of an association of *FLT3* gene expression and mRNA transcripts encoding HSP110, HSP90, and BIM (p=0,00011-0,016). Positive R values indicate positive coregulation of gene expression; negative R values indicate negative coregulation of gene expression; TPM, transcripts per million reads. The graphs show the Spearman correlation coefficients. Such correlation coefficients describe monotonic correlations between two sets of data (Rovetta A., Cureus. 2020; Hauke J., Kossowski T., Quaestiones Geographicae 2011). GEPIA2 uses the non-log scale for calculation and use the log-scale axis for visualization.


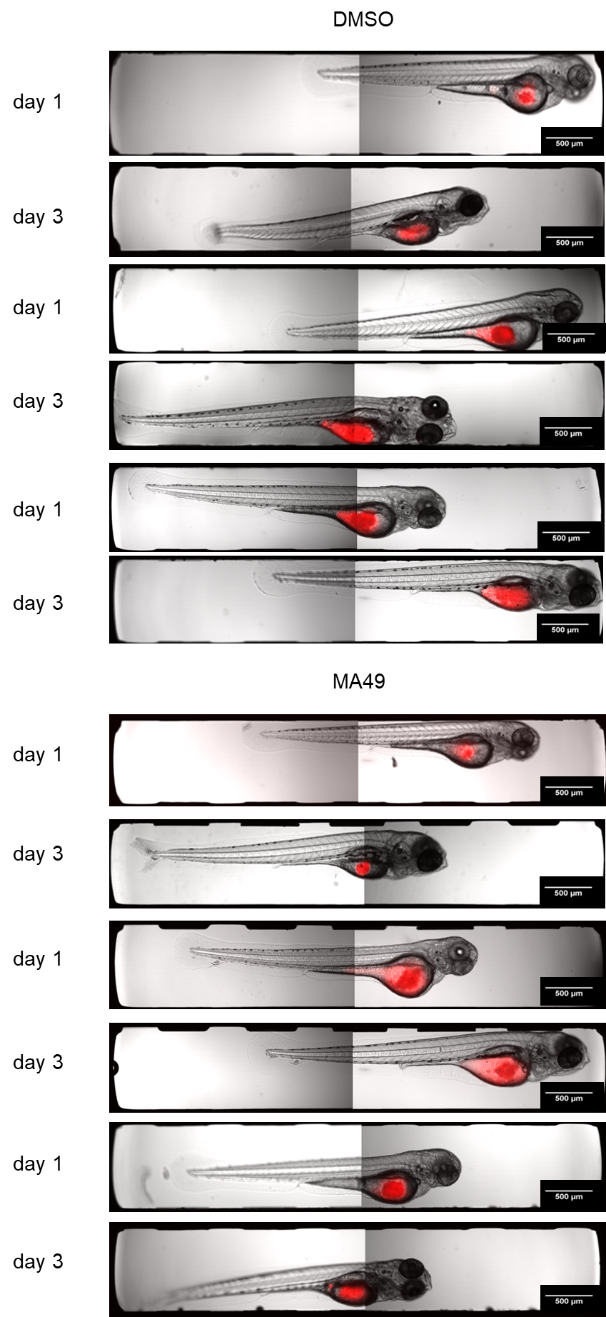


**Figure S5. MA49 in zebrafish xenograft larvae.** Representative images of zebrafish larvae injected with a suspension of fluorescently labeled MV4-11 cells, tumor cells appear red. Larvae were imaged using confocal microscopy 24 h after the injection and before the treatment (day 1) and 48 h after the treatment with 200 nM MA49 or DMSO used as a solvent (day 3). Fluorescent and bright fields are merged.
