# Supplementary material for: Selective degradation of mutant FMS-like tyrosine kinase-3 requires BIM-dependent depletion of heat shock proteins
Source: Leukemia. 2024 Sep 17;38(12):2561–72. doi: 10.1038/s41375-024-02405-5 (PMC11588663; doi:10.1038/s41375-024-02405-5)
Supplement: Supplementary file 2 — Chemical synthesis and verifications [file 41375_2024_2405_MOESM2_ESM.pdf]

Chemical synthesis

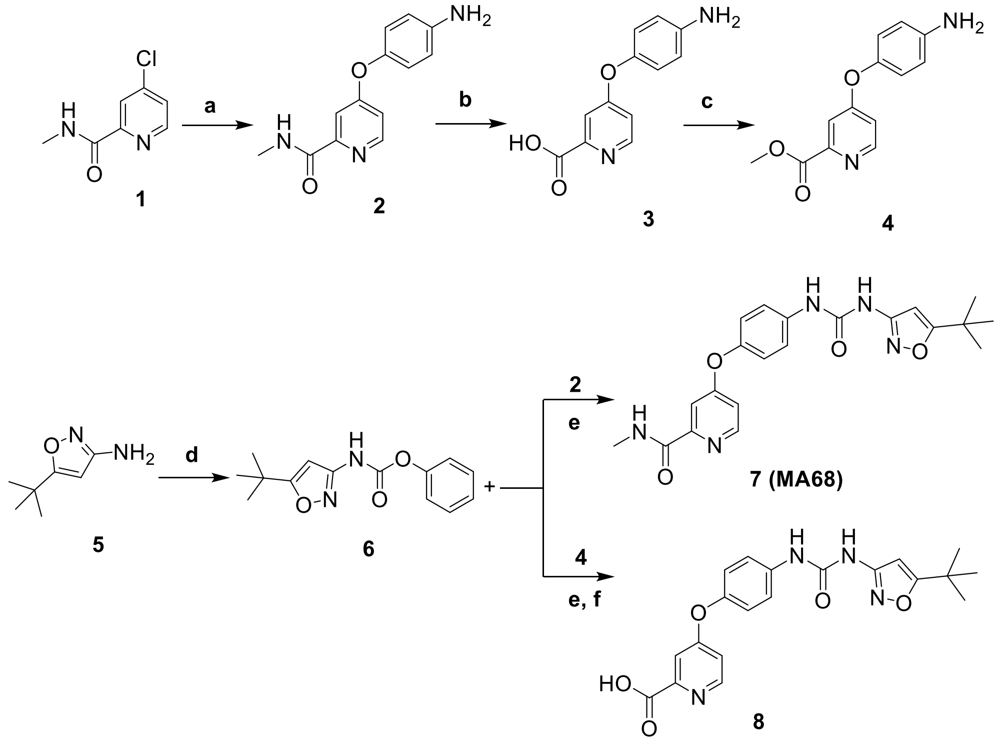

Scheme 1: Synthesis of the FLT3 inhibitor **7** (MA68) and the carboxylic acid derivative **8** used for PROTAC synthesis. Reagents and conditions: (a) *t*-BuOK, K_2CO_3, DMF 80 ^{0}C, 3 h; (b) KOH, reflux, 5 h; (c) $SOCl_2$, MeOH, reflux, 8 h; (d) phenyl chloroformate, Pyridine, THF, RT, 3 h; (e) DMAP, DMF, 50 ^{0}C, overnight; (f) LiOH. H_2O, THF, H_2O, RT, 3 h.

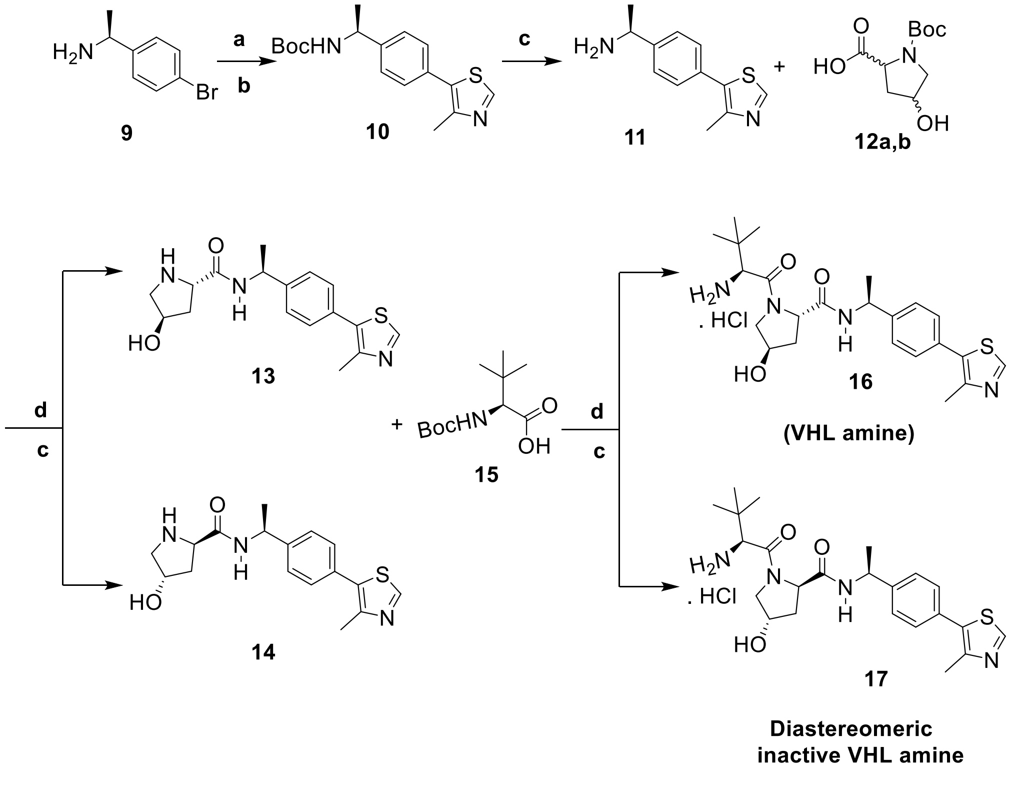

Scheme 2: Synthesis of the VHL amine building block **16** and its negative control **17**. Reagents and conditions: (a) Boc_2O, $NaHCO_3$, 2 h; (b) 4-methylthiazole, $Pd(OAc)_2$, KOAc, dimethylacetamide, 90 °C, 12 h; (c) 4 N HCl/dioxane, 0 °C to RT, 16h; (d) HATU, DIPEA, DCM, 0 °C to RT, 16 h.

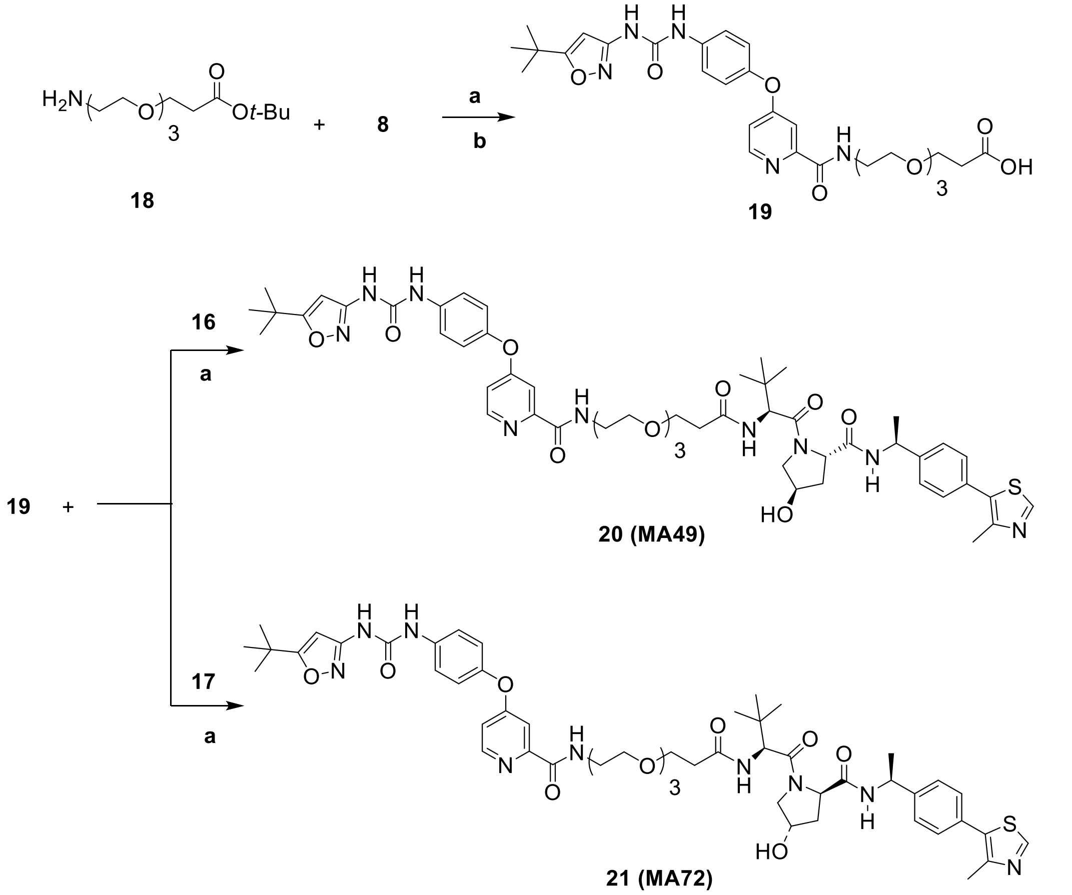

Scheme 3: Synthesis of the VHL-based PROTAC **20** (MA49) and the negative control PROTAC **21** (MA72). Reagents and conditions: (a) HATU, DIPEA, DMF, RT, 4 h; (b) TFA, DCM, 0 °C to RT, 1 h.

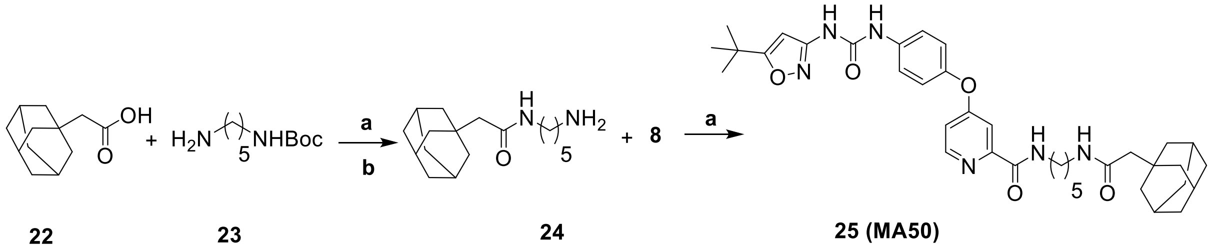

Scheme 4: Synthesis of the hydrophobic-tag degrader **25** (MA50). Reagents and conditions: (a) HATU, DIPEA, DMF, RT, 4 h; (b) TFA, DCM, 0 ˚C to RT, 30 min.

Synthesis of compounds

General methods

All materials and reagents were purchased from Sigma-Aldrich Co., Ltd. (Darmstadt, Germany) and abcr GmbH (Karlsruhe, Germany). All solvents were analytically pure. Thin-layer chromatography was carried out on aluminium sheets coated with silica gel 60 F254 (Merck,Darmstadt, Germany). For medium-pressure liquid chromatography (MPLC), Biotage SNAP ultra-HP-sphere 25 µm columns containing silica gel were used. Dichloromethane (DCM): methanol (MeOH) and n-heptane: ethyl acetate mixtures were used as elution systems for MPLC. Purity was measured by UV absorbance at 254 nm. The HPLC consisted of a LiChrosorb® RP-18 (5 µm) 100-4.6 Merck column (Merck, Darmstadt, Germany), two LC-10AD pumps, a SPD-M10A VP PDA detector, and a SIL-HT autosampler, all from the manufacturer Shimadzu (Kyoto, Japan). The absorption spectra were recorded with a SPD-M10A diode array detector Shimadzu spectrophotometer (Kyoto, Japan). Mass spectrometry analyses were performed with a Finnigan MAT710C (Thermo Separation Products, SanJose, CA, USA) for the ESI MS spectra and with a LTQ (linear ion trap) Orbitrap XL hybrid mass spectrometer (Thermo Fisher Scientific, Bremen, Germany) for the HRMS-ESI (high-resolution mass spectrometry) spectra. For the HRMS analyses, the signal for the isotopes with the highest prevalence was given and calculated. ^{1}H NMR spectra were taken on a Varian Inova 400 using deuterated DMSO as solvent. Chemical shifts were referenced to the residual solvent signals. The following abbreviations and formulas for solvents and reagents were used: ethyl acetate (EtOAc), N,N-dimethylformamide (DMF), dimethyl sulfoxide (DMSO), methanol (MeOH), tetrahydrofuran (THF), water (H_2O), dichloromethane (DCM), N,N-diisopropylethylamine (DIPEA), O-(7-azabenzotriazol-1-yl)-*N*,*N*,*N'*,*N'*-tetramethyluronium-hexafluorphosphate (HATU) and hydrochloric acid (HCl), trifluoroacetic acid (TFA).

General synthetic procedures

Method I: Amide coupling.

A solution of the appropriate carboxylic acid derivative (1.0 equiv.), HATU (1.0 equiv.) and DIPEA (5.0 equiv.) in DMF (5 mL) was stirred at RT for 10 min., then the corresponding amine derivative (1.1 equiv.) was added. The reaction mixture was stirred at RT for 4 h. After completion of the reaction as indicated by TLC, water was added, and the mixture was extracted using ethyl acetate. The combined organic layer was washed with an aqueous 1 M ammonium chloride solution, followed by an aqueous 1 M sodium bicarbonate solution and brine. The combined organic extract was dried over anhydrous sodium sulfate, the organic layer was filtered, then evaporated under reduced pressure to yield the crude amide, which was purified using MPLC using DCM : MeOH (3-10% MeOH).

Method II: *N*-Boc-deprotection or *tert*-butyl ester hydrolysis.

The appropriate *N*-Boc-protected amine derivative or *tert*-butyl ester was dissolved at 0 °C in dry DCM (5 mL), and then TFA (5 mL) was added. The reaction mixture was stirred at RT for 30–60 min. The solvent was evaporated to dryness to provide the corresponding amine or the carboxylic acid derivative

Intermediates **2-4** were synthesized as previously reported [1].

Synthesis and characterization of intermediates and final compounds

Phenyl (5-(tert-butyl)isoxazol-3-yl)carbamate (6).

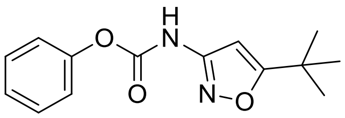

To a solution of 3-amino-5-tert-butylisoxazole (**5**) (0.5 g, 3.5 mmol. 1 equiv.) in THF (15 mL) and pyridine (0.6 mL, 7.4 mmol, 2.1 equiv.), phenyl chloroformate (0.47 mL, 3.7 mmol, 1.05 equiv.), was added dropwise over 15 min at 0°C. Then, the reaction mixture was stirred at RT for 3 h. Water (50 mL) was added, and the mixture was extracted with DCM (3*50 mL). The combined organic layers were dried over sodium sulfate, filtered, and evaporated under reduced pressure to obtain the titled compound as a white semi-solid in a quantitative yield,

which was used for the next step without further purification. MS m/z: 283.34 [M + Na]$^+$. ^{1}H NMR (400 MHz, DMSO-d$_6$) δ 11.12 (s, 1H), 7.45 – 7.37 (m, 2H), 7.29 – 7.16 (m, 3H), 6.41 (s, 1H), 1.27 (s, 9H).

4-(4-(3-(5-(*tert*-Butyl)isoxazol-3-yl)ureido)phenoxy)-*N*-methylpicolinamide 7 (MA68).

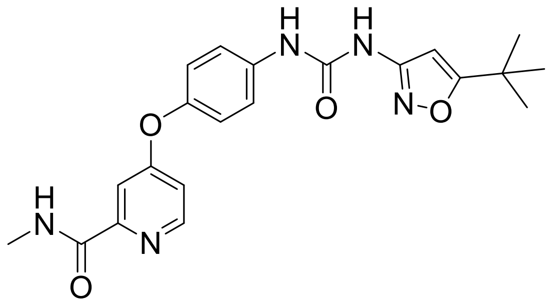

To a stirred solution of intermediate **2** (1.0 equiv.) and 4-dimethylaminopyridine (DMAP) (2.4 equiv.) in DMF (10 mL), intermediate **5** (1.1 equiv.) was added. The reaction mixture was stirred at 50 ºC overnight and cooled to RT. Water (20 mL) was added, and the mixture was extracted with ethyl acetate (3*50mL). The combined organic layer was washed with brine, dried over sodium sulfate, filtered, and evaporated under reduced pressure. The obtained residue was purified by MPLC using ethyl acetate 70-80% in *n*-heptane and was obtained as a white solid. (yield: 72%). ^{1}H NMR (400 MHz, DMSO-d$_6$) δ 9.51 (s, 1H), 8.92 (s, 1H), 8.78 – 8.66 (m, 1H), 8.47 (d, *J* = 5.6 Hz, 1H), 7.61 – 7.49 (m, 2H), 7.36 (d, *J* = 2.6 Hz, 1H), 7.19 – 7.04 (m, 3H), 6.48 (s, 1H), 2.76 (d, *J* = 4.9 Hz, 3H), 1.27 (s, 9H). ^{13}C NMR (101 MHz, DMSO-d$_6$) δ 180.6, 166.4, 164.2, 158.8, 152.9, 151.9, 150.8, 148.4, 137.2, 121.9, 120.8, 114.5, 109.2, 92.9, 32.9, 28.8, 26.4. HRMS m/z: 410.182 [M+H]$^+$; calculated $C_{21}H_{24}O_4N_5^+$: 410.1828. HPLC: rt 11.83 min (purity 98.9%).

4-(4-(3-(5-(*tert*-Butyl)isoxazol-3-yl)ureido)phenoxy)picolinic acid (8)

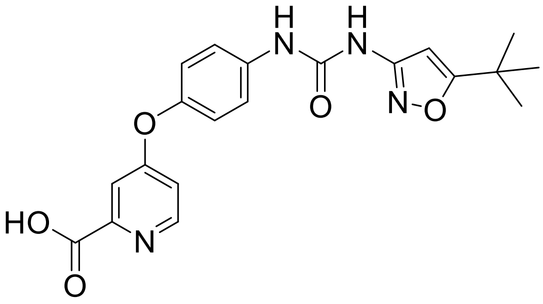

Intermediate **8** was synthesized in two-steps synthetic procedure. First, intermediate **4** and **6** were reacted using the same procedure as described for compound **7**, then the obtained intermediate (1.0 equiv.) was stirred in a mixture of THF: H_2O (1:1) (10 mL) containing LiOH.H_2O (5.0 equiv.). The mixture was stirred at RT for 5 h and the pH value of the solution was adjusted to 5. The resulting solid was filtrated and washed with water to provide the corresponding carboxylic acid **8** as a white solid, (yield: 90%). MS m/z: 395.20 [M-H]$^-$. ^{1}H NMR (400 MHz, DMSO-d_6) δ 9.92 (s, 1H), 9.48 (s, 1H), 8.49 (d, *J* = 5.6 Hz, 1H), 7.61 –7.54 (m, 2H), 7.38 (t, *J* = 4.5 Hz, 1H), 7.18 – 7.07 (m, 3H), 6.49 (s, 1H), 1.28 (s, 9H).

The VHL building blocks **16**, **17** and the PEG linker **18** were prepared following the previously reported procedure [2] and [3] respectively.

1-(4-(4-(3-(5-(*tert*-Butyl)isoxazol-3-yl)ureido)phenoxy)pyridin-2-yl)-1-oxo-5,8,11-trioxa-2-azatetradecan-14-oic acid (19)

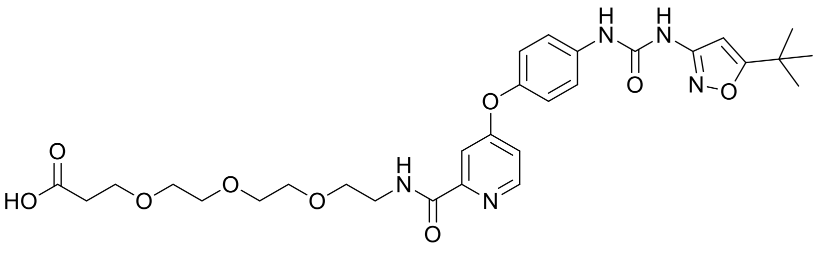

Intermediate **19** was synthesized through the amide coupling reaction between intermediates **8** and **18** following the general procedure as described in method I, followed by the *tert*-butyl ester hydrolysis using method II to get intermediate **19** as a white solid (yield: 52% over 2 steps). MS m/z: 598.41 [M-H]$^-$. ^{1}H NMR (500 MHz, DMSO-d_6) δ 12.09 (s, 1H), 9.61 (s, 1H),

9.06 (s, 1H), 8.67 (t, *J* = 5.9 Hz, 1H), 8.54 – 8.45 (m, 1H), 7.59 – 7.54 (m, 2H), 7.38 (d, *J* = 2.5 Hz, 1H), 7.20 – 7.11 (m, 3H), 6.49 (s, 1H), 3.69 – 3.40 (m, 14), 2.44 – 2.37 (m, 2H), 1.28 (s, 9H).

4-(4-(3-(5-(*tert*-Butyl)isoxazol-3-yl)ureido)phenoxy)-*N*-((*S*)-14-((2*S*,4*R*)-4-hydroxy-2-(((*S*)-1-(4-(4-methylthiazol-5-yl)phenyl)ethyl)carbamoyl)pyrrolidine-1-carbonyl)-15,15-dimethyl-12-oxo-3,6,9-trioxa-13-azahexadecyl)picolinamide 20 (MA49)

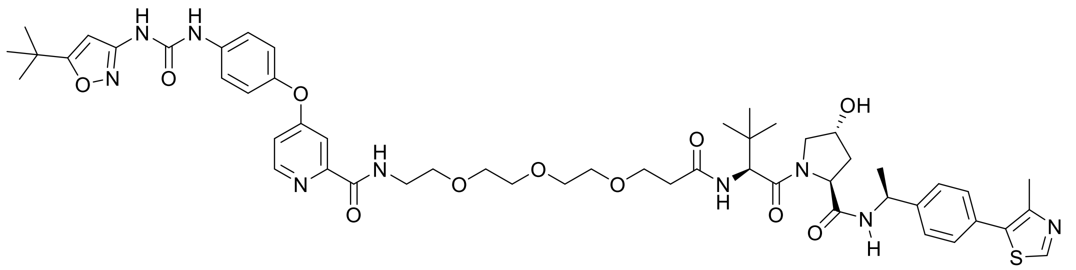

Compound **MA49** was synthesized through the amide coupling between intermediates **16** and **19** using method I and the product was obtained as a white solid (yield: 56%). ^{1}H NMR (400 MHz, DMSO-d$_6$) δ 9.54 (s, 1H), 9.01 (s, 1H), 8.96 (s, 1H), 8.67 (t, *J* = 5.8 Hz, 1H), 8.49 (dd, *J* = 5.7, 2.5 Hz, 1H), 8.34 (d, *J* = 7.8 Hz, 1H), 7.82 (d, *J* = 9.3 Hz, 1H), 7.59 – 7.53 (m, 2H), 7.43 – 7.33 (m, 5H), 7.19 – 7.11 (m, 3H), 6.49 (s, 1H), 5.07 (d, *J* = 3.5 Hz, 1H), 4.94 – 4.85 (m, 1H), 4.50 (d, *J* = 9.3 Hz, 1H), 4.41 (t, *J* = 8.0 Hz, 1H), 4.25 (s, 1H), 3.63 – 3.39 (m, 16H), 2.54 – 2.49 (m, 1H), 2.44 (s, 3H), 2.37 – 2.28 (m, 1H), 2.03 – 1.95 (m, 1H), 1.77 (ddd, *J* = 12.9, 8.4, 4.6 Hz, 1H), 1.35 (d, *J* = 7.0 Hz, 3H), 1.29 – 1.23 (m, 9H), 0.90 (s, 9H). HRMS m/z: 1048.458 $[M+Na]^+$; calculated $C_{52}H_{67}O_{11}N_9NaS^+$: 1048.4578. HPLC: rt 12.10 min (purity 99.14%).

4-(4-(3-(5-(*tert*-Butyl)isoxazol-3-yl)ureido)phenoxy)-*N*-((*S*)-14-((2*R*,4*S*)-4-hydroxy-2-(((*S*)-1-(4-(4-methylthiazol-5-yl)phenyl)ethyl)carbamoyl)pyrrolidine-1-carbonyl)-15,15-dimethyl-12-oxo-3,6,9-trioxa-13-azahexadecyl)picolinamide 21 (MA72).

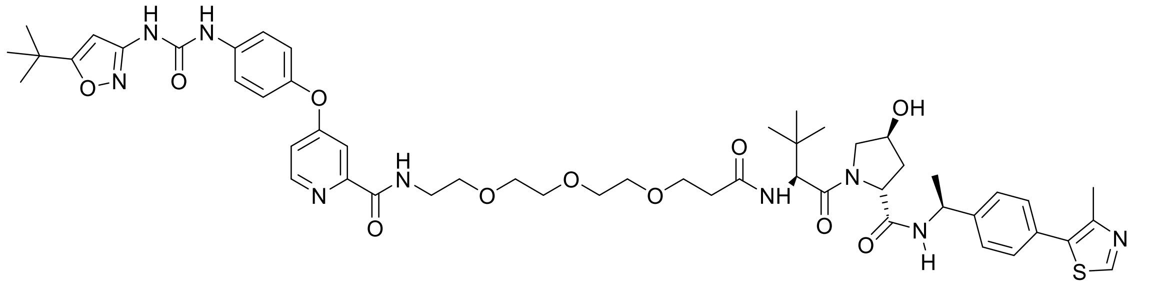

Compound **MA72** was synthesized through the amide coupling between intermediates **17** and **19** using method I and the product was obtained as a white solid (yield: 50%). ^{1}H NMR (400 MHz, DMSO-d$_6$) δ 9.53 (s, 1H), 9.00 – 8.90 (m, 2H), 8.65 (t, *J* = 5.7 Hz, 1H), 8.48 (d, *J* = 5.6 Hz, 1H), 8.00 (d, *J* = 8.0 Hz, 1H), 7.94 (d, *J* = 7.8 Hz, 1H), 7.56 (d, *J* = 8.9 Hz, 2H), 7.48 – 7.33 (m, 5H), 7.19 – 7.09 (m, 3H), 6.49 (s, 1H), 5.08 (d, *J* = 3.8 Hz, 1H), 4.94 – 4.83 (m, 1H), 4.42 – 4.33 (m, 2H), 4.29 (d, *J* = 3.9 Hz, 1H), 3.78 (dd, *J* = 10.4, 5.3 Hz, 1H), 3.60 – 3.36 (m, 15H), 2.59 – 2.51 (m, 1H), 2.42 (s, 3H), 2.31 – 2.22 (m, 1H), 2.03 – 1.87 (m, 2H), 1.33 – 1.18 (m, 12H), 0.94 (s, 9H). HRMS m/z: 1048.458 [M+Na]$^+$; calculated $C_{52}H_{67}O_{11}N_9NaS^+$: 1048.4578. HPLC: rt 15.12 min (purity 94.07%).

5-(2-(Adamantan-1-yl)acetamido)pentan-1-aminium 2,2,2-trifluoroacetate (**24**)

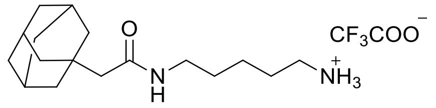

Intermediates **22** and **23** were reacted through the amide coupling reaction according to method I, and the obtained intermediate was subjected to *N*-Boc deprotection according to method II to obtain intermediate **24** as a colorless oil (yield: 65% over 2 steps). MS m/z: 279.26 [M+H]$^+$. ^{1}H NMR (400 MHz, $CDCl_3$) δ 7.94 (s, 1H), 6.62 – 6.56 (m, 3H), 3.21 (t, *J* = 10.7 Hz, 2H), 3.03 – 2.88 (m, 2H), 2.84 (s, 1H), 2.03 – 1.89 (m, 5H), 1.80 – 1.32 (m, 17H).

***N*-(5-(2-(Adamantan-1-yl)acetamido)pentyl)-4-(4-(3-(5-(*tert*-butyl)isoxazol-3-yl)ureido)-phenoxy)picolinamide 25 (MA50)**

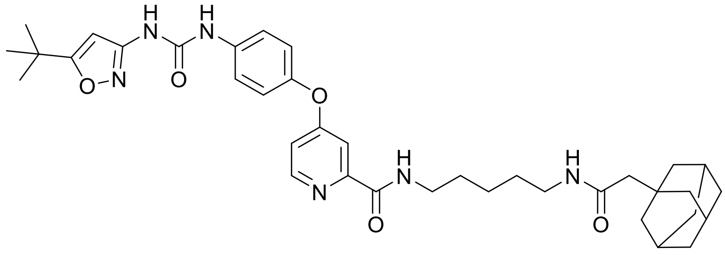

Compound **MA50** was obtained through the amide coupling reaction between intermediates **8** and **24** following method I and the product was obtained as a white solid (yield: 60%). ^{1}H NMR (400 MHz, DMSO-d_6) δ 9.52 (s, 1H), 8.94 (s, 1H), 8.71 (t, *J* = 6.0 Hz, 1H), 8.47 (d, *J* = 5.6 Hz, 1H), 7.60 – 7.51 (m, 3H), 7.35 (d, *J* = 2.6 Hz, 1H), 7.19 – 7.08 (m, 3H), 6.47 (s, 1H), 3.22 (dd, *J* = 13.3, 6.7 Hz, 2H), 2.96 (dd, *J* = 12.7, 6.8 Hz, 2H), 1.84 (s, 3H), 1.75 (s, 2H), 1.59 (d, *J* = 11.9 Hz, 3H), 1.54 – 1.41 (m, 10H), 1.40 – 1.30 (m, 2H), 1.30 – 1.14 (m, 12H). ^{13}C NMR (101 MHz, DMSO-d_6) δ 180.6, 170.1, 166.4, 163.6, 158.8, 152.9, 151.9, 150.7, 148.4, 137.2, 121.9, 120.8, 114.5, 109.2, 92.9, 50.5, 42.6, 38.7, 36.9, 32.9, 32.6, 29.29, 29.25, 28.8, 28.5, 24.3. HRMS m/z: 679.358 $[M+Na]^+$; calculated $C_{37}H_{48}O_5N_6Na^+$: 679.3584. HPLC: rt 12.95 min (purity 96.58%).

Analytical data for the final compounds

¹HNMR of MA68

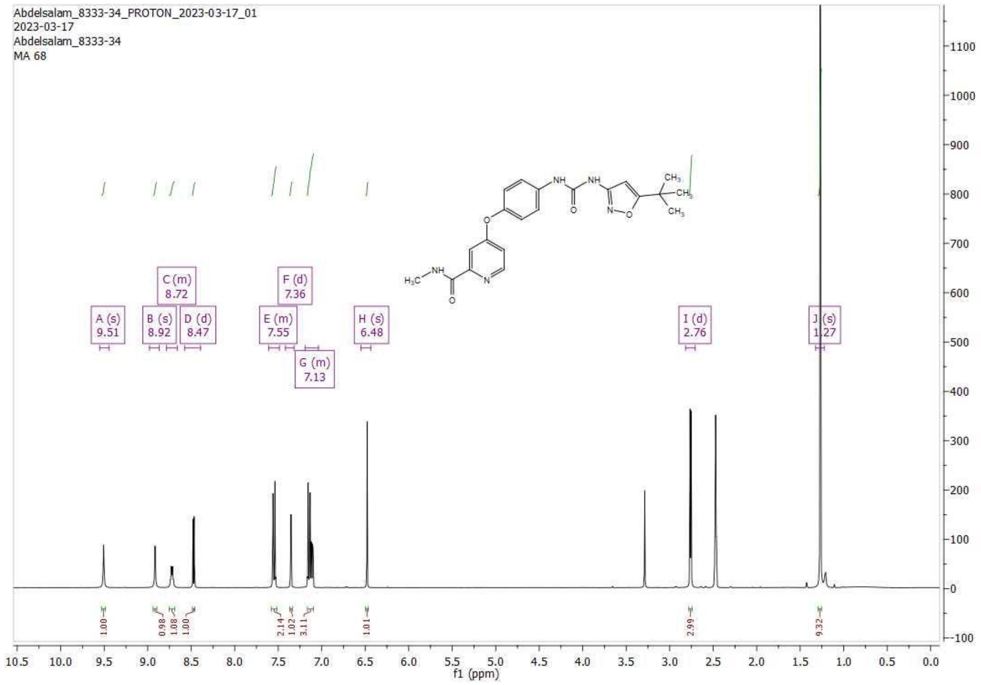

¹³CNMR of MA68

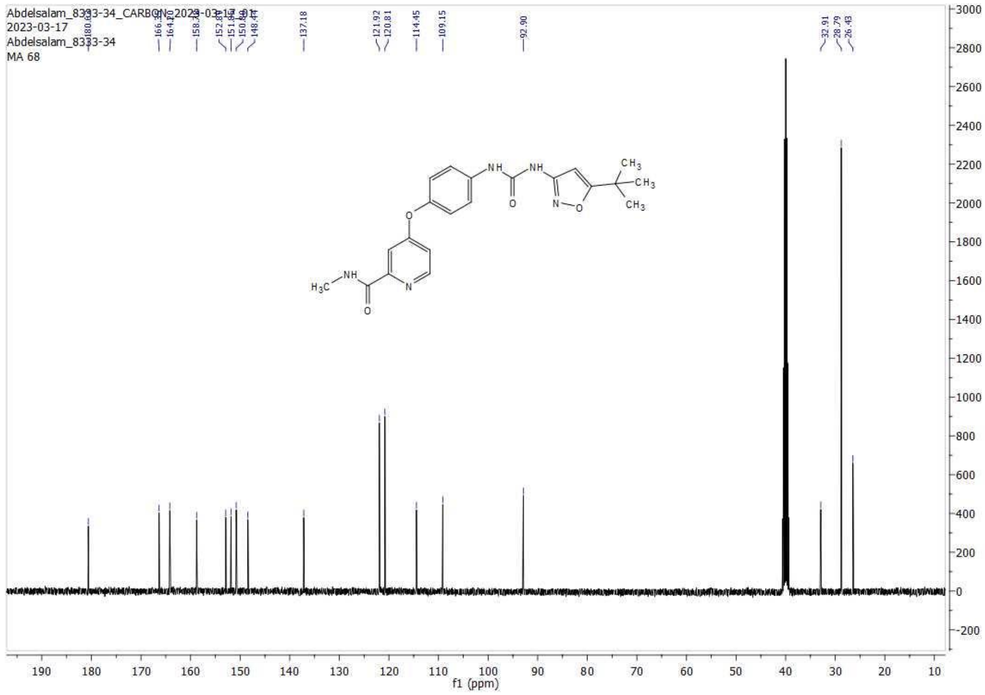

HRMS of MA68

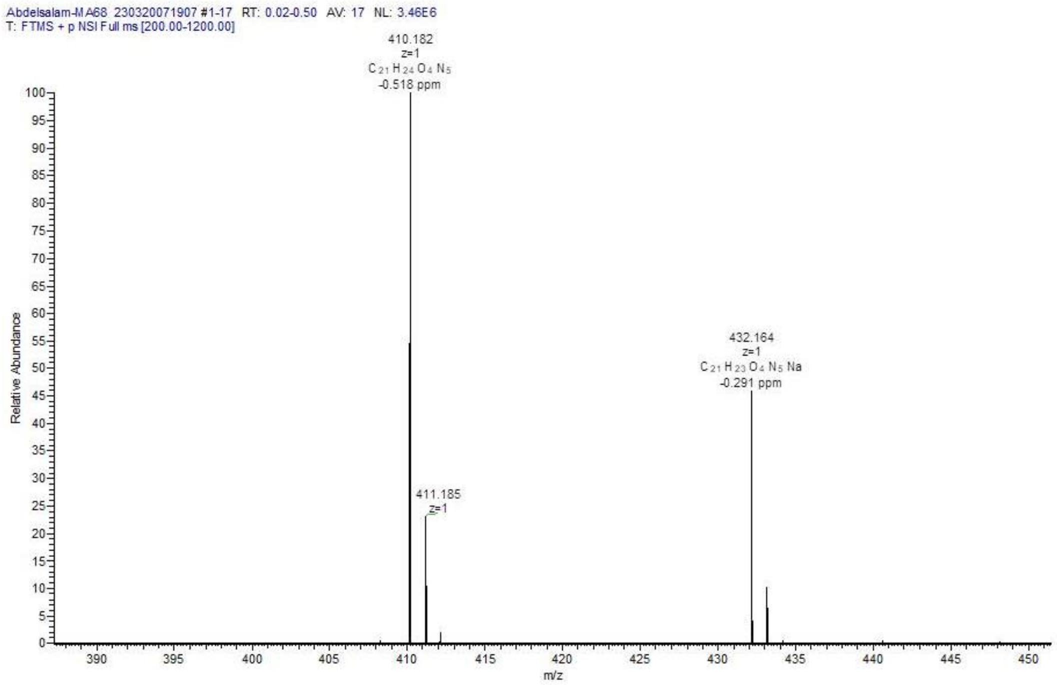

HPLC of MA68

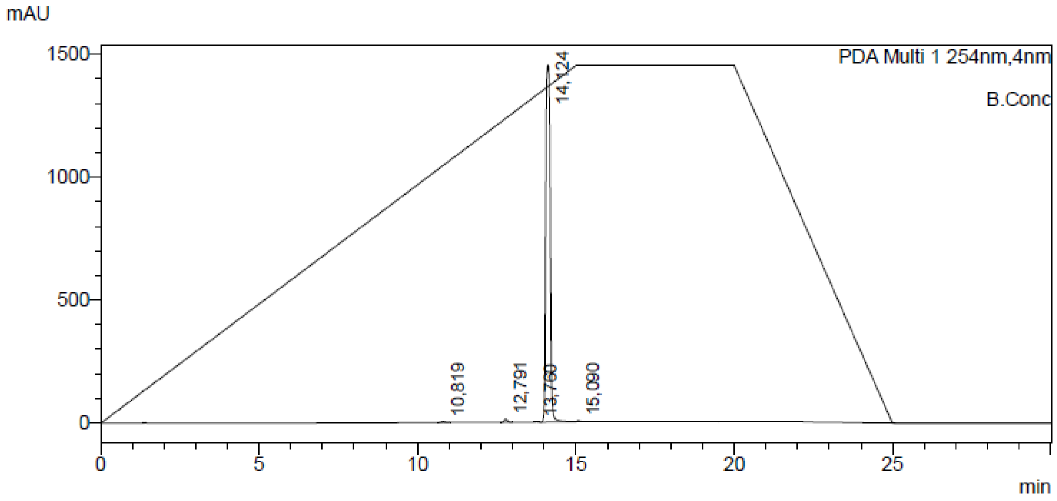

PDA Ch1 254nm

Peak#	Ret. Time	Area	Height	Area%
1	10,819	34893	5089	0,255
2	12,791	85093	13905	0,623
3	13,760	6419	1144	0,047
4	14,124	13517982	1449506	98,898
5	15,090	24283	4489	0,178
Total		13668670	1474133	100,000

^{1}HNMR of MA49

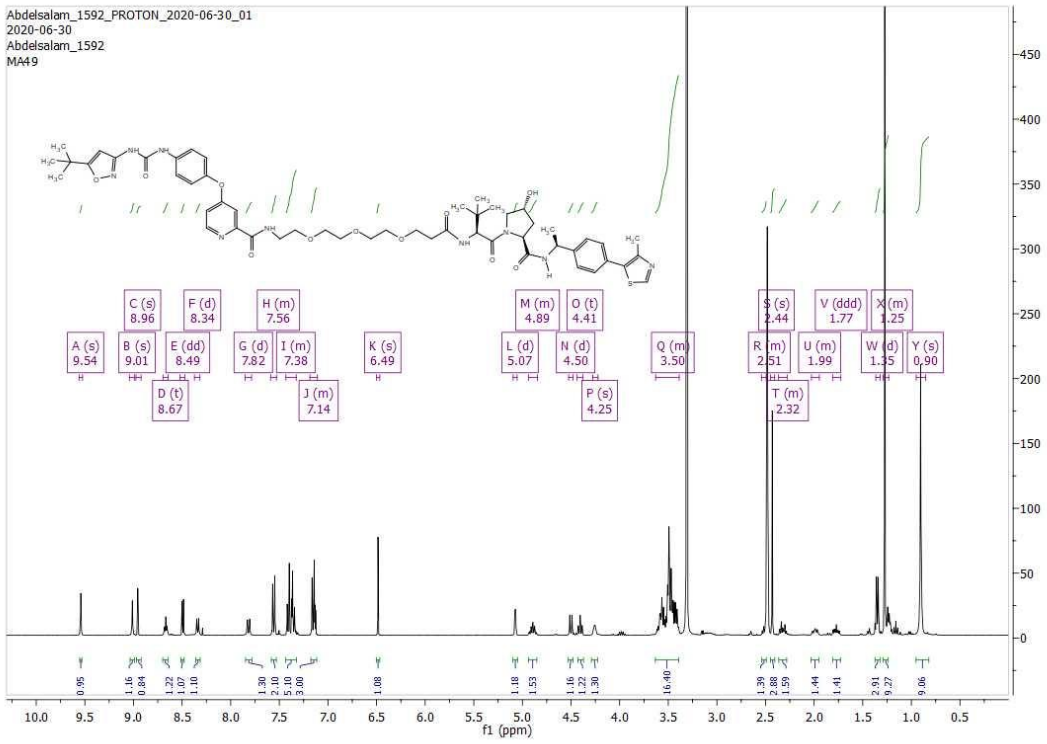

HRMS of MA49

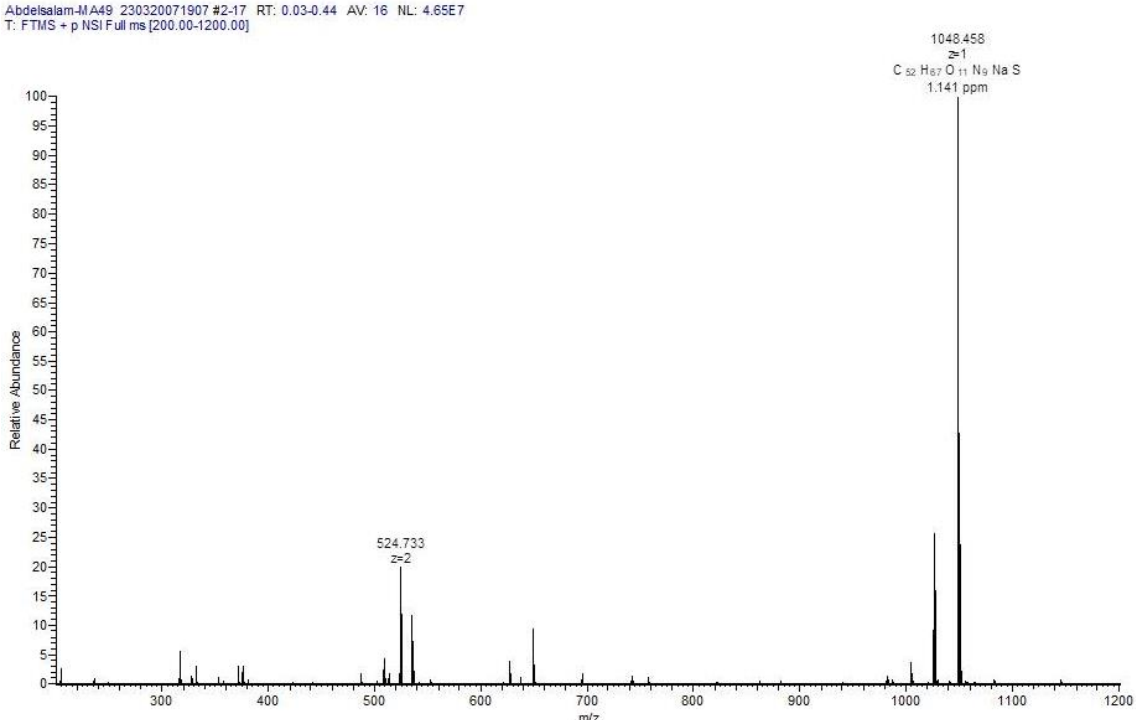

HPLC of MA49

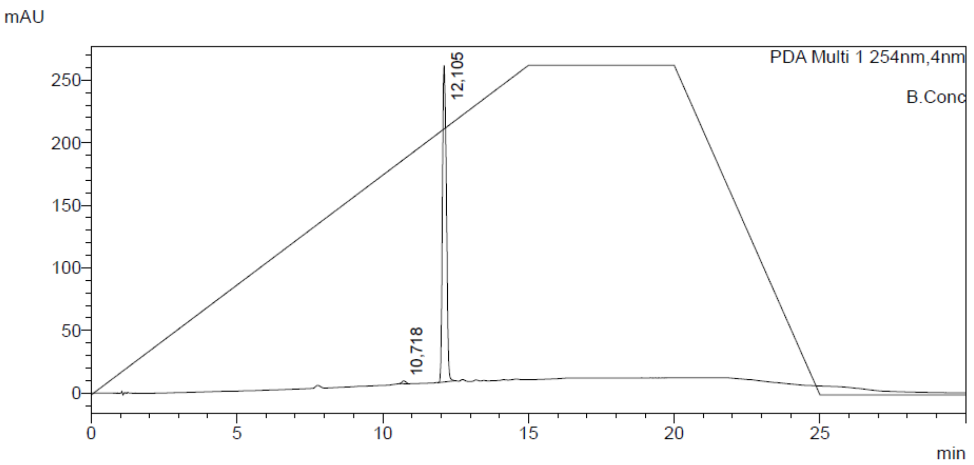

PDA Ch1 254nm

Peak#	Ret. Time	Area	Height	Area%
1	10,718	20641	2247	0,860
2	12,105	2379079	252890	99,140
Total		2399720	255137	100,000

^{1}HNMR of MA72

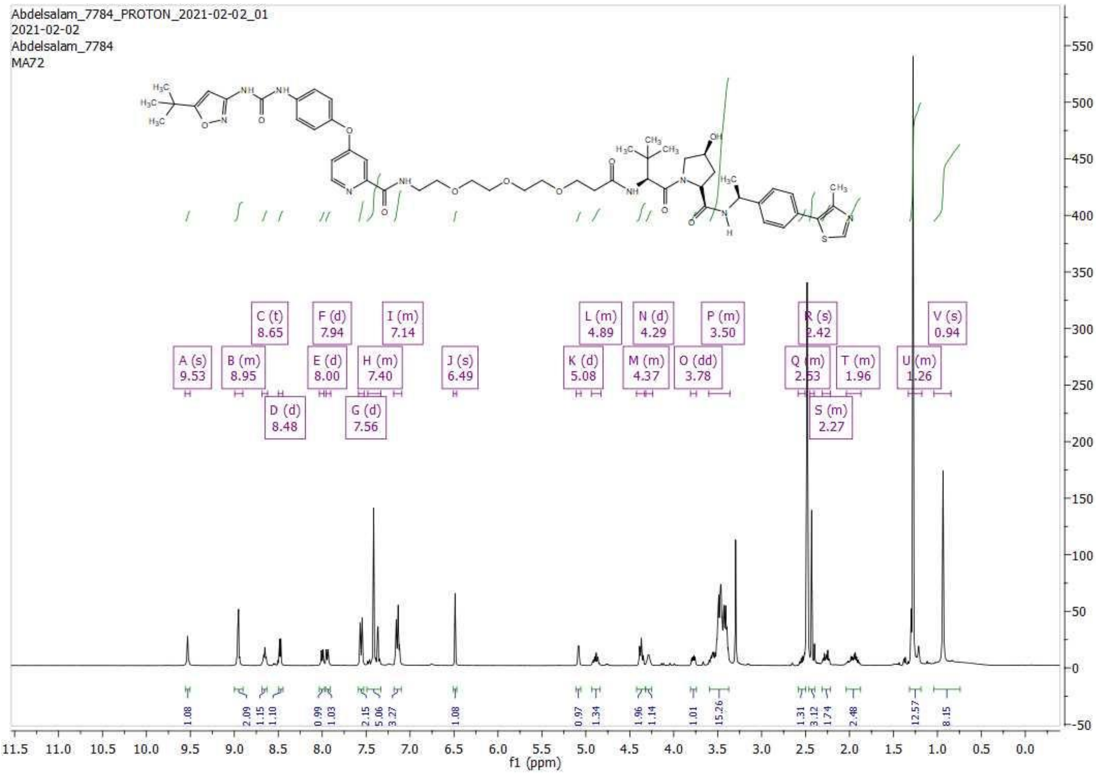

HRMS of compound MA72

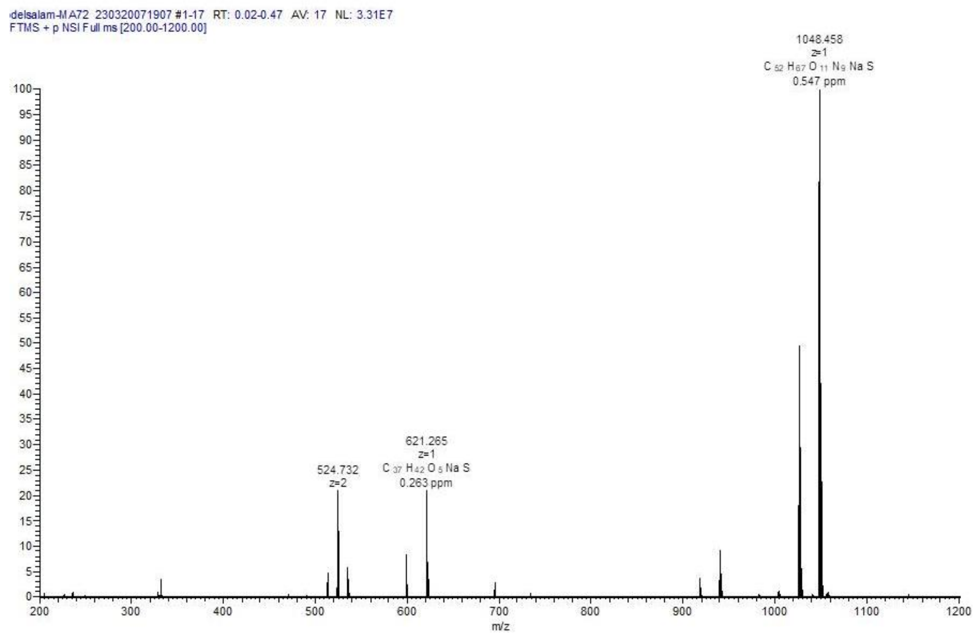

HPLC of MA72

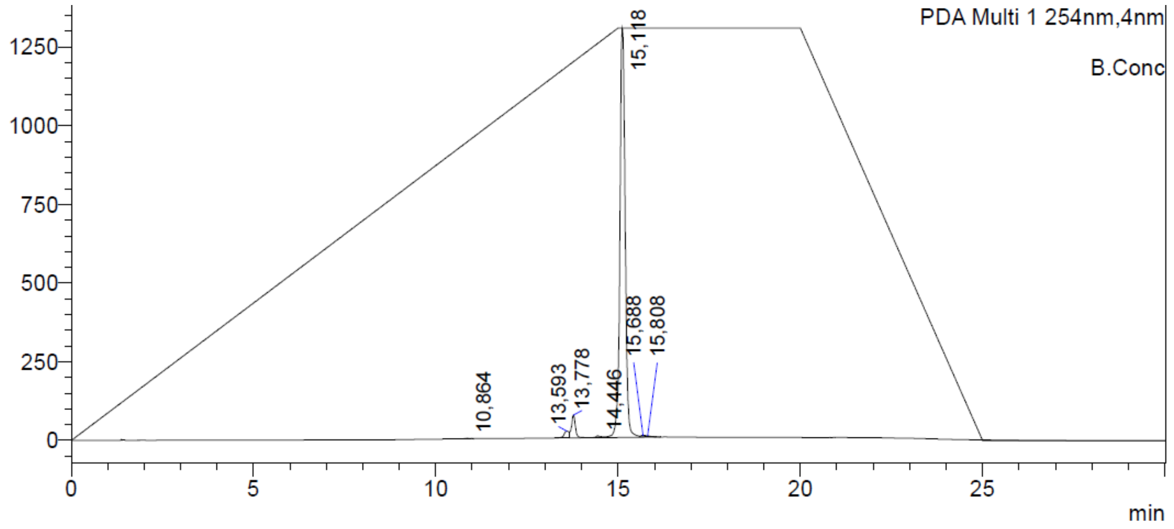

PDA Ch1 254nm

Peak#	Ret. Time	Area	Height	Area%
1	10,864	8150	1175	0,058
2	13,593	207457	21841	1,476
3	13,778	540971	73165	3,848
4	14,446	45626	5474	0,325
5	15,118	13224860	1300521	94,070
6	15,688	16730	2704	0,119
7	15,808	14666	1799	0,104
Total		14058459	1406681	100,000

^{1}H NMR of MA50

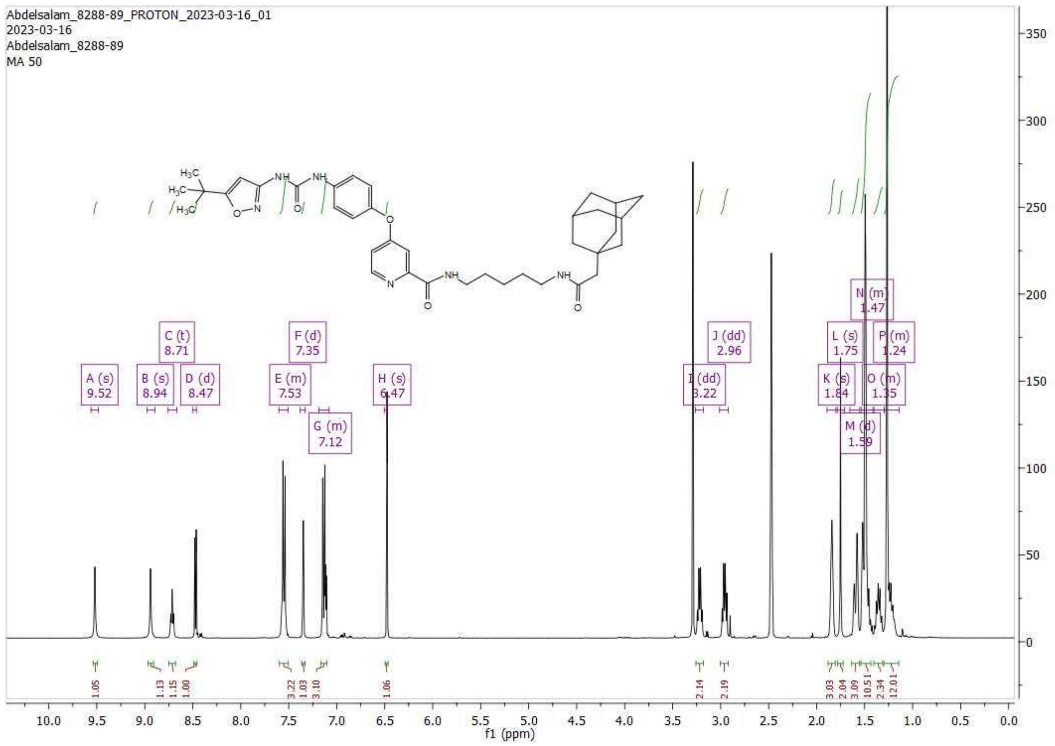

^{13}C NMR of MA50

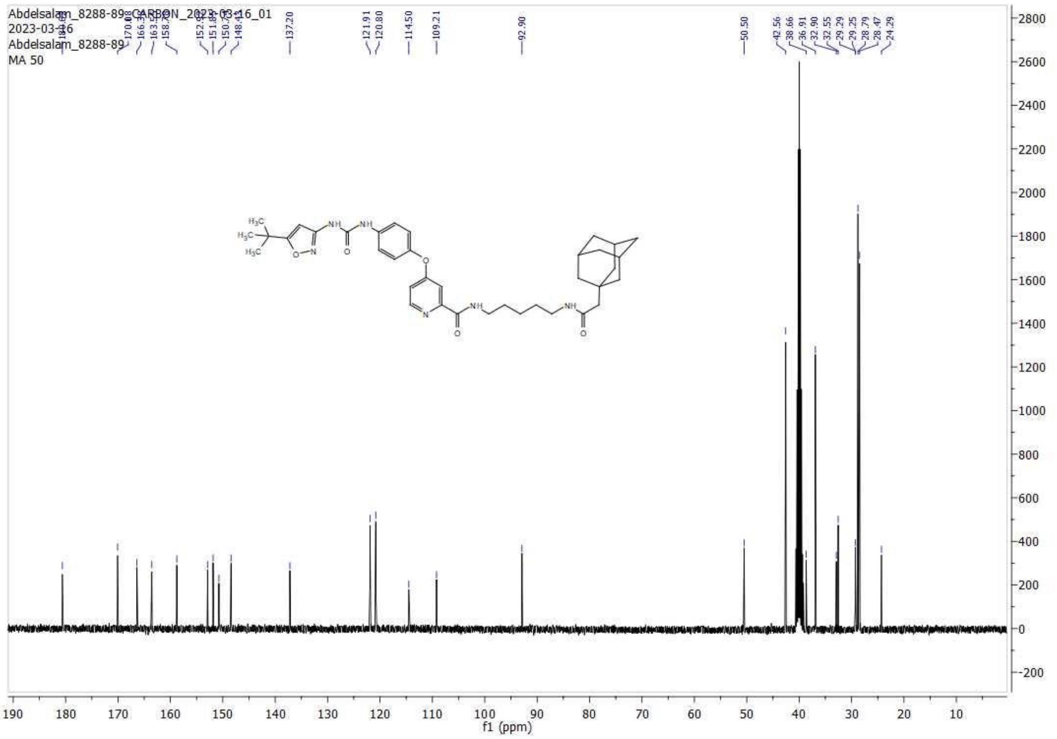

HRMS of MA50

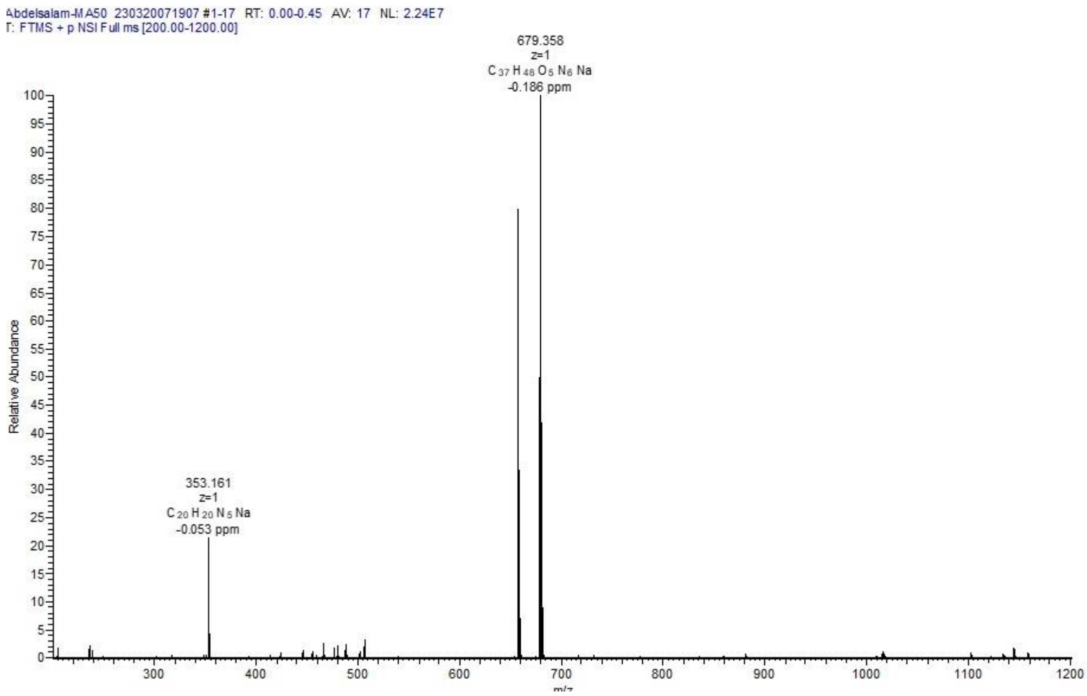

HPLC of MA50

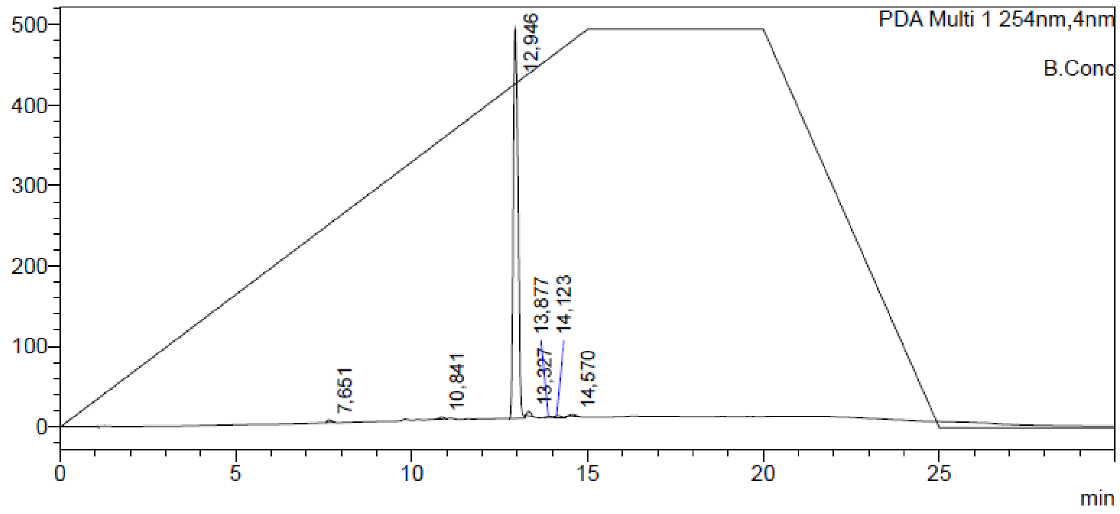

PDA Ch1 254nm

Peak#	Ret. Time	Area	Height	Area%
1	7,651	26161	3014	0,580
2	10,841	25052	2697	0,556
3	12,946	4354709	484454	96,587
4	13,327	39871	5667	0,884
5	13,877	16726	1487	0,371
6	14,123	22922	2005	0,508
7	14,570	23131	2017	0,513
Total		4508572	501339	100,000

References

1. K. Wang, K. Kuerbana, Q. Wan, Z. Yu, L. Ye, and Y. Chen. Introduction of Mercaptoethyl at Sorafenib Pyridine-2-Amide Motif as a Potentially Effective Chain to Further get Sorafenib-PEG-DGL. Molecules 2020, 25(3), 573.
2. J. Hu, B. Hu, M. Wang, F. Xu, B. Miao, CY. Yang, M. Wang, Z. Liu, D. F. Hayes, K. Chinnaswamy, J. Delproposto, J. Stuckey, and S. Wang. Discovery of ERD-308 as a Highly Potent Proteolysis Targeting Chimera (PROTAC) Degrader of Estrogen Receptor (ER). J. Med. Chem. 2019, 62, 1420−1442.
3. L. Peng, Z. Zhang, C. Lei, S. Li, Z. Zhang, X. Ren, Y. Chang, Y. Zhang, Y. Xu, and K. Ding. Identification of New Small-Molecule Inducers of Estrogen-related Receptor α (ERRα) Degradation. ACS Med. Chem. Lett. 2019, 10, 5, 767–772.
