# Supplementary material for: Selective degradation of mutant FMS-like tyrosine kinase-3 requires BIM-dependent depletion of heat shock proteins
Source: Leukemia. 2024 Sep 17;38(12):2561–72. doi: 10.1038/s41375-024-02405-5 (PMC11588663; doi:10.1038/s41375-024-02405-5)
Supplement: Supplementary file 3 — Supplementary information File - Materials and Methods, Halilovic et al. [file 41375_2024_2405_MOESM3_ESM.docx]

**Supplementary information File - Materials and Methods, Halilovic et al.**

**Materials and Methods**

**Protein lysates, immunoblot, densitometry, and antibodies**

Cells were harvested on ice and centrifuged at 300xg for 5 min. Pellets were washed with ice-cold PBS, centrifuged (300xg/5 min), and lysed in NET-N buffer (100 mM NaCl, 10 mM Tris-HCl pH 8,1 mM EDTA, 10% glycerin, 0.5% NP-40; plus complete protease inhibitor tablets (Roche, Mannheim, Germany) and phosphatase inhibitor cocktail 2 (Sigma Aldrich, Munich, Germany)) for 25 min on ice, sonicated (10 s/20% amplitude) and centrifuged (18,800xg/25 min/4°C). Protein concentrations of these whole cell lysates were measured by Bradford assay. Proteins were detected by SDS-PAGE and immunoblotting using the quantitative Odyssey Infrared Imaging System (Licor, Bad Homburg, Germany) or enhanced chemoluminescence (GE Healthcare, Freiburg, Germany) with Western Lighting Plus-ECL substrate (PerkinElmer, Waltham, USA). ImageJ was used to perform densitometric analyses. Antibodies were anti-FLT3 (ab245116, 1:300), anti-AKT (ab32505, 1:2000), anti-BIM (ab32158, 1:5000), anti-phospho-PERK (Thr982) (ab192591, 1:1000), anti-alpha Tubulin (ab176560, 1:2000) from Abcam, Cambridge, UK; anti-phospho-FLT3 (Tyr591) (3461, 1:1000), anti-cleaved caspase-3 (Asp175) (9661, 1:1000), anti-phospho-Akt (Ser473) (9271, 1:1000), anti-phospho-c-Kit (Tyr719) (3391, 1:1000), anti-BiP (3177, 1:1000), anti-PERK (3192, 1:1000), anti-CHOP (2895, 1:1000) from Cell Signalling Technology, Leiden, Netherlands; anti-STAT5 (33-5900, 1:1000), anti-phospho-STAT5 (Tyr694) (MA5-14973, 1:1000), anti-VHL (PA5-27322, 1:1000) from Thermo Fisher, Dreieich, Germany; anti-HSP27 (sc-13132, 1:1000), anti-HSP90 alpha/beta (sc-13119, 1:1000), anti-HSP105 (sc-74550, 1:1000), anti-HSP70 (sc-66048, 1:1000), anti-HSP60 (sc-13115, 1:1000), anti-IRE1α (sc-390960, 1:500), anti-ATF6α (sc-166659, 1:250), anti-beta-actin (sc-47778, 1:1000), vinculin (sc-73614, 1:1000), GAPDH (sc-32233, 1:2000) from Santa Cruz, Heidelberg, Germany; anti-PARP1 (556362, 1:500) from BD Pharmingen, Heidelberg, Germany. Secondary antibodies: HRP-coupled anti-mouse (7076; 1:2500) and anti-rabbit (7074; 1:2500) were purchased from Cell Signalling Technology, Leiden, Netherlands. IRDye® 680RD- (mouse: 925–68070; 1:10.000; rabbit: 925-68071; 1:10.000) or IRDye® 800CW-coupled secondary antibodies (mouse: 925-32210, 1:10.000; rabbit: 925-32211, 1:10.000) were from Licor, Bad Homburg, Germany.

**Cell lines**

MV4-11 (from a 10-year-old boy), MOLM-13 (from a 20-year-old male), RS4-11 (from a 32-year-old woman), and HMC1.2 (from a male mast cell leukemia patient) cells were gifts from Prof. Frank Böhmer or Dr. Sebastian Drube, University of Jena, Germany (originally from the DSMZ, Braunschweig, Germany), and hTERT-immortalized retinal pigment epithelial RPE1 cells were given by Prof. Thomas Hofmann, University Medical Center Mainz, Germany. Leukemia cells were authenticated by DNA fingerprinting (DNA profiling using eight different and highly polymorphic short tandems repeats) at the Leibniz-Institute (DSMZ, Braunschweig, Germany. Cells were maintained at 37°C and 5% CO_2_ in a humidified atmosphere. Growth medium for MV4-11 and MOLM-13 cell lines was RPMI-1640, HMC1.2 cells were cultured in Iscove's Modified Dulbecco's Medium (IMDM), RPE1 cells were cultured in Dulbecco's Modified Eagle Medium (DMEM). Media were supplemented with 10% fetal bovine serum (FBS) and 1% penicillin/streptomycin (Sigma Aldrich, Munich, Germany). Cell lines were confirmed to be mycoplasma-free by MycoStrip (Invivogen, Toulouse France).

**Peripheral blood mononuclear cells (PBMCs), murine bone marrow stem cells, and their differentiation into macrophages and dendritic cells**

PBMCs and SCA-1/c-KIT-double positive stem cells were collected and analyzed as recently described by us^1,2^. In brief, murine bone marrow cells were isolated from three 12-week old male and female, sacrificed mice. The cells were treated with 50-100 nm MA49 or MA68 for 24 h. The next day, cells were harvested, washed, and stained with c-KIT, SCA-1, and a cocktail of lineage markers (CD3, CD4, CD8, NK1.1, Gr-1, CD19, CD11b). Lineage negative cells were gated for SCA-1+c-KIT+ double positive cells and viability was assessed by fixable viability dye 780 (FVD, negative staining indicates living cells). Additionally, bone marrow cells were seeded in 12-well plates at a density of 200,000 cells/ml and cultured for 7 days in culture medium supplemented with either 10 ng/ml recombinant mGM-CSF to yield bone marrow-derived dendritic cells (BMDCs) or 10 ng/mL recombinant mM-CSF to yield bone marrow-derived macrophages (BMDMs). After plating, the cells were treated with 50-100 nM of either MA68 or MA49. Culture medium and inhibitors were replenished on days 3 and 6 of culture. After one week, cells were harvested, washed, and stained with CD86 PE (GL1, BD,#553692), CD11c PE-Cy7 (N418,eBioscience, #25-0114-81), and F4/80 FITC (BM8, eBioscience, #11-4801-85). Viability was assessed by fixable viability dye 506 (FVD- cells). After debris and doublet exclusion, total cell count was assessed and FVD- (viable) cells were further gated for CD11c or F4/80 and the mean fluorescence intensity of CD86 was assessed.

**Primary AML cells and patient characteristics**

These are AML 1: female, 80 years, FLT3 WT, ASXL1mut, SRF2mut 46 XY; AML 2: male 25 years, FLT3-ITD, normal cytogenetics; AML 3: male, 78 years, FLT3-ITD, NPM1/TKD WT, normal cytogenetics. Mononuclear cells were separated by Ficoll-Hypaque density gradient and subsequent dose response assays set up with 7.5x10^5^ cells/ml primary AML cells cultured in IMDM medium supplemented with 200 mM L-glutamine. Cells were harvested at 72 h for Cell Titer Glo cytotoxicity assays (Promega, Hampshire, UK). Luminescence was detected using a chameleon V plate reader (Hidex). Calcusyn version 2.1 (Biosoft, Cambridge, UK) and used to calculate EC_50_ responses.

**Inhibitors and chemicals**

MA49, MA50, MA68, and MA72 were synthesized by us (**schemes 1-4**, **supplementary data file SI**). Analytical characterization of the compounds and determination of their purity is described in **supplementary data file SI**. Annexin-V-FITC-conjugate was from Miltenyi Biotec, Bergisch Gladbach, Germany; propidium iodide, tunicamycin, and cycloheximide from Sigma-Aldrich, Munich, Germany; Onalespib (AT13387) from Selleckchem, Cologne Germany.

**Kinase binding assay**

Dissociation constants (K_d_) for MA49, MA50, and MA68 at human FLT3-ITD were determined by KINOMEscan^TM^ (Eurofins DiscoverX Corporation, San Diego, CA, USA).

**Detection of apoptosis by annexin V/PI staining**

Cells were collected 24-72 h after drug treatment, washed with 1x PBS, and stained with annexin V-FITC (Miltenyi Biotec, Bergisch Glabdach, Germany). After 15 min, cells were stained with PI (50 µg/ml). Flow cytometry was performed with a FACS Canto II (BD Bioscience, Heidelberg, Germany). The software tool FACSDiva 7.0 was used to evaluate the data.

**Transfections with siRNAs**

MV4-11 cells were seeded in RPMI medium containing 10% FBS without antibiotics one day before electroporation. Cells were harvested and centrifuged (200xg, 5 min), the supernatant was aspirated, and cells were washed with PBS. After centrifugation (200xg, 5 min) and aspiration of PBS, cells were resuspended in 100 mL buffer R (MPK10096, Thermo Fisher, Dreieich, Germany) and siRNA solutions were added. Cells were electroporated with 1.350 V, 1 pulse for 35 milliseconds, using the Neon™ Transfection System (MPK10096, Thermo Fisher, Dreieich, Germany), and then transferred in a prewarmed culture medium. Knockdown of BIM (encoded on the *BCL2L11* mRNA) and VHL (encoded on the *VHL* mRNA) in MV4-11 cells was performed by transfecting 100 pmol SMARTpool ON-TARGETplus siRNA against *BCL2L11* (L-004383-00-0005, Dharmacon, Cambridge, UK), 100 pmol Silencer Select siRNA against *VHL* (s14789, Thermo Fisher, Dreieich, Germany), or non-targeting control siRNA (sc-37007; sc-44321, Santa Cruz, Heidelberg, Germany). After 24 h, cells were treated. Efficient knockdown was confirmed by immunoblotting.

**Zebrafish lines, embryo xenotransplantation, and treatment**

The zebrafish (*Danio rerio*) embryo xenograft experiment was performed in analogy to a previously described protocol^3^, with the following specifications. MV4-11 cells at density of 1×10^6^ viable cells/ml were stained with 5 µl CM-DiD (Thermo Fisher Scientific) solution and injected into the yolk sac of zebrafish wild-type AB line embryos on day 2 post fertilization. Next day, xenotransplanted embryos bearing labeled MV4-11 cells were treated with 200 nM MA49 or the respective solvent control. Animals were randomly distributed into treatment or control groups. Tumor growth was assessed using an ImageXpress Confocal High-Content Imaging System (Molecular Devices), both before drug exposure (day 1) and 48 hours (day 3) after treatment. The tumor volume was quantified unbiasedly with a semi-automated macro for ImageJ, as previously described^3^. Tumor growth was measured by calculating volume differences [%] between day 1 and day 3 imaging points. To determine the responses to the drug, the zebrafish-adapted Response Evaluation Criteria in Solid Tumors (RECIST) was used^3^.

**Gene Expression Profiling Interactive Analysis version 2 (GEPIA2) analyses**

This resource for gene expression data relies on 84 cancer subtypes from the databases [TCGA](https://www.cancer.gov/about-nci/organization/ccg/research/structural-genomics/tcga) and [GTEx](https://gtexportal.org/home/) and comprises gene expression quantification according to RNA-sequencing^4^.

**Statistical analyses**

Statistics were performed with GraphPad Prism 6. Significant differences between groups were determined with one-way or two-way ANOVA. Multiple comparisons were done with Bonferroni correction. Asterisks indicate p values (*p, <0.05; **p, <0.01; ***p, <0.001; ****p, <0.0001). Error bars represent standard error of the mean (SEM).

1 Zeyn, Y. *et al.* Histone deacetylase inhibitors modulate hormesis in leukemic cells with mutant FMS-like tyrosine kinase-3. *Leukemia* **37**, 2319-2323, doi:10.1038/s41375-023-02036-2 (2023).

2 Hieber, C. *et al.* Inhibitors of the tyrosine kinases FMS-like tyrosine kinase-3 and WEE1 induce apoptosis and DNA damage synergistically in acute myeloid leukemia cells. *Biomed Pharmacother* **177**, 117076, doi:10.1016/j.biopha.2024.117076 (2024).

3 Seiboldt, T. *et al.* Synergy of retinoic acid and BH3 mimetics in MYC(N)-driven embryonal nervous system tumours. *Br J Cancer*, doi:10.1038/s41416-024-02740-5 (2024).

4 Tang, Z., Kang, B., Li, C., Chen, T., Zhang, Z. GEPIA2: an enhanced web server for large-scale expression profiling and interactive analysis. *Nucleic Acids Re* **47(W1)**, 556-560, doi:10.1093/nar/gkz430 (2019).
