# Supplementary figures and images for: Selective degradation of mutant FMS-like tyrosine kinase-3 requires BIM-dependent depletion of heat shock proteins
Source: Leukemia. 2024 Sep 17;38(12):2561–72. doi: 10.1038/s41375-024-02405-5 (PMC11588663; doi:10.1038/s41375-024-02405-5)

Original immunoblots


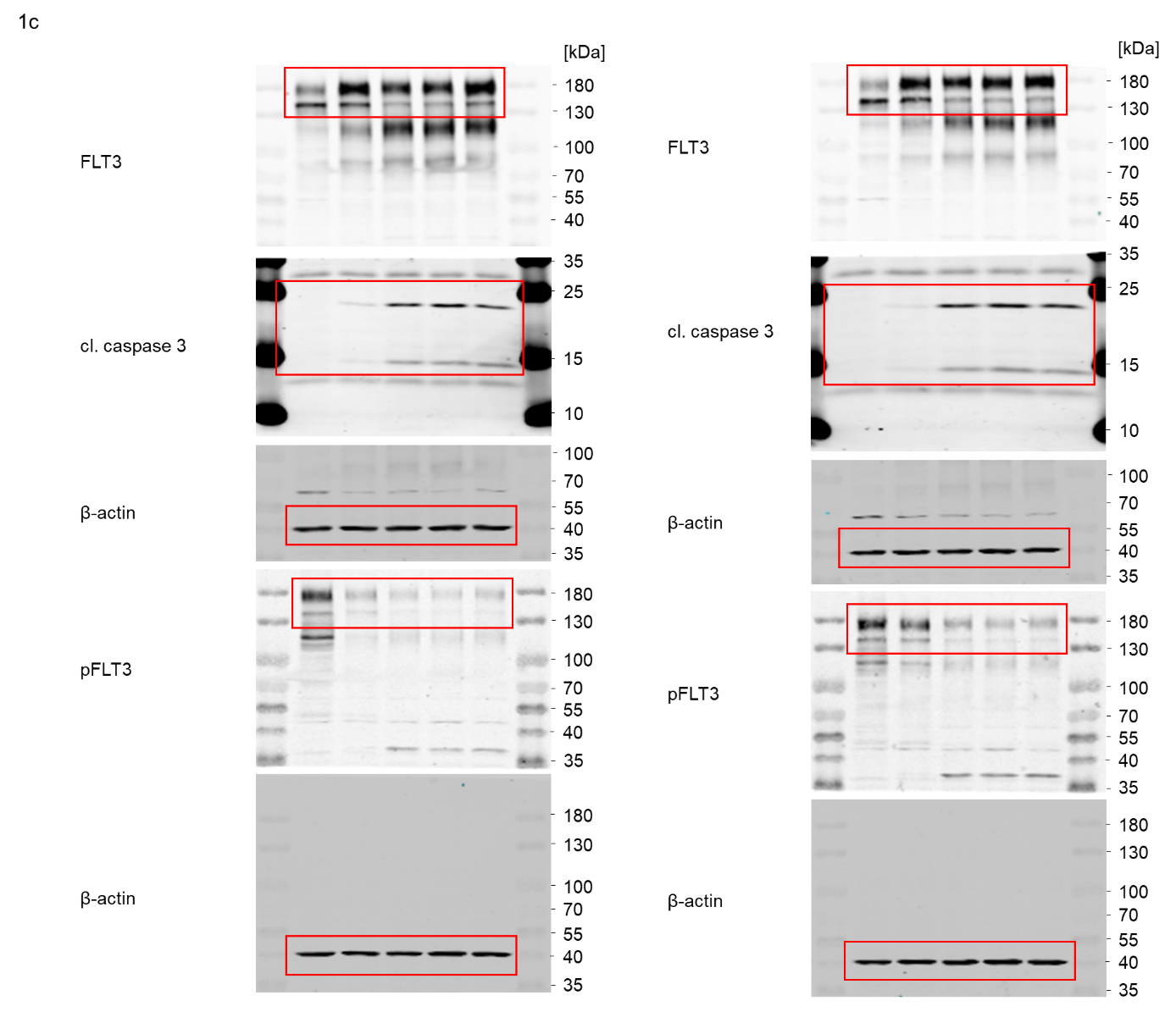


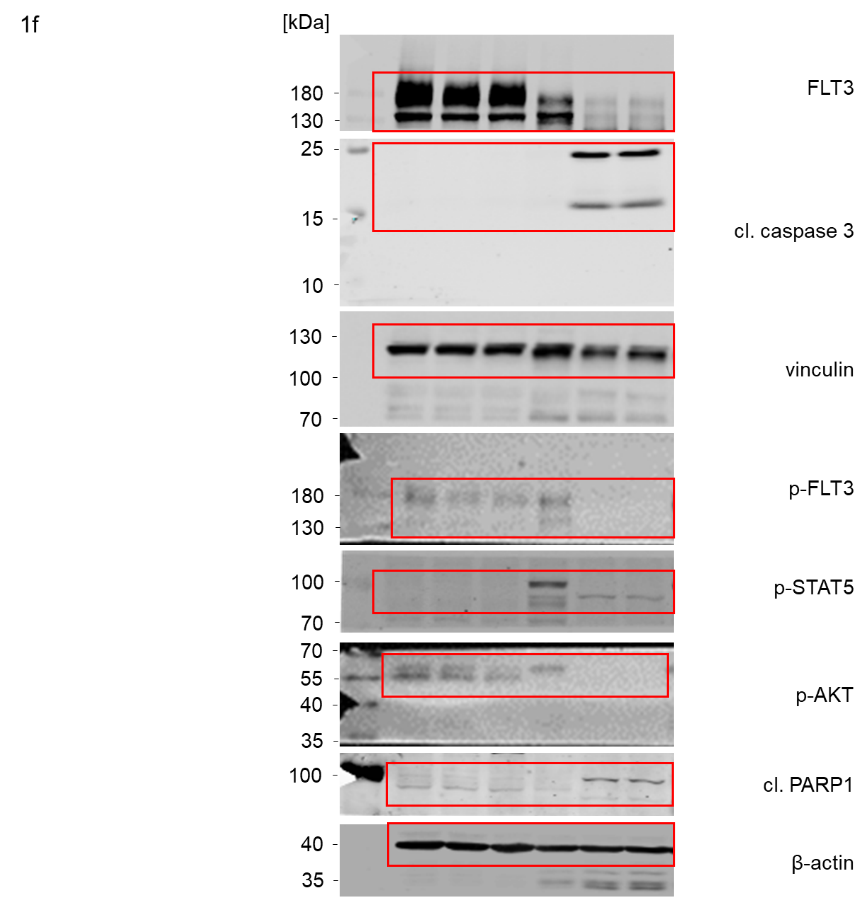


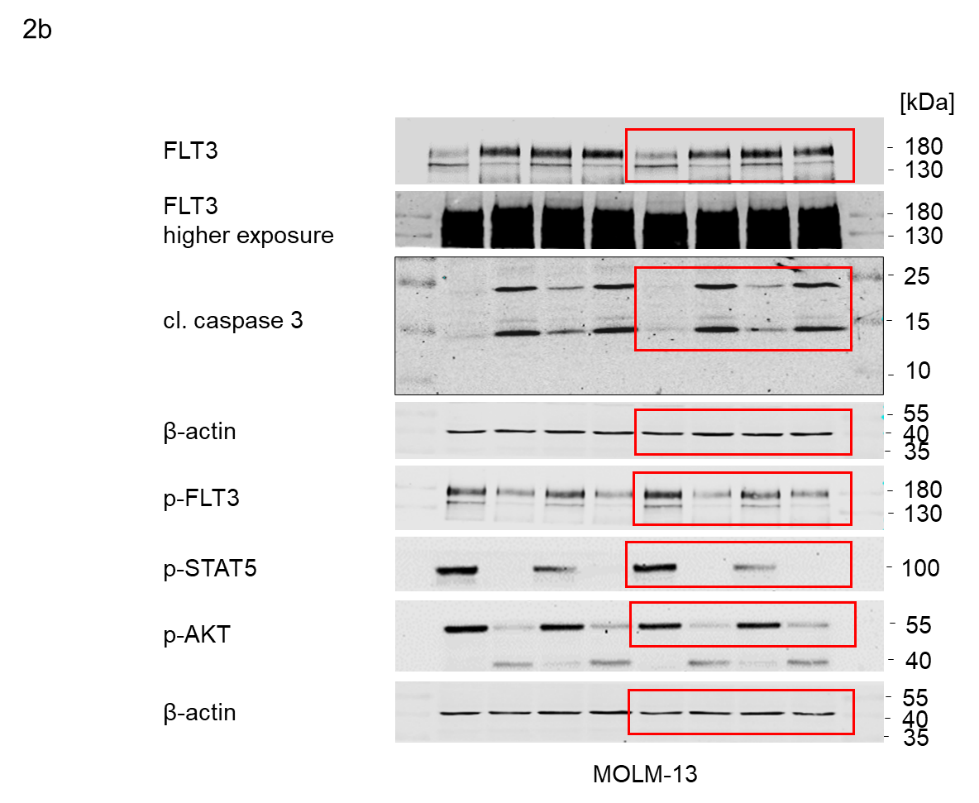


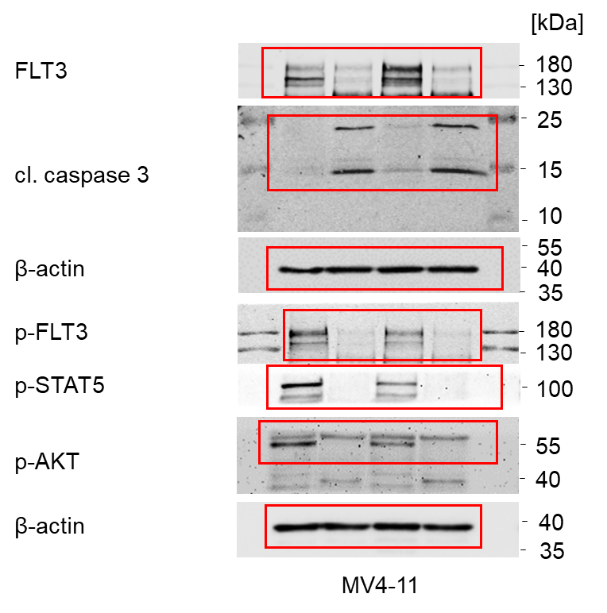


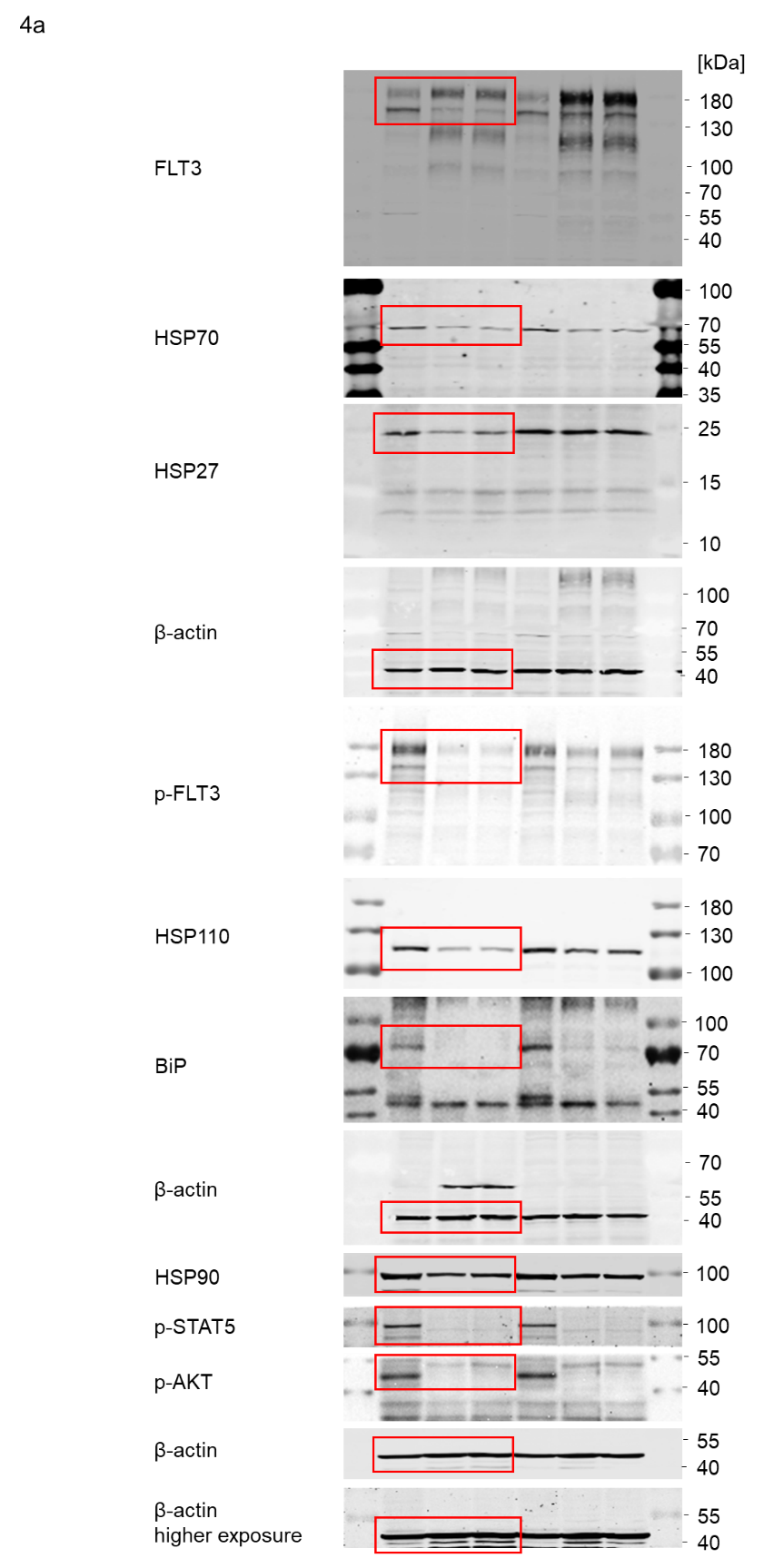


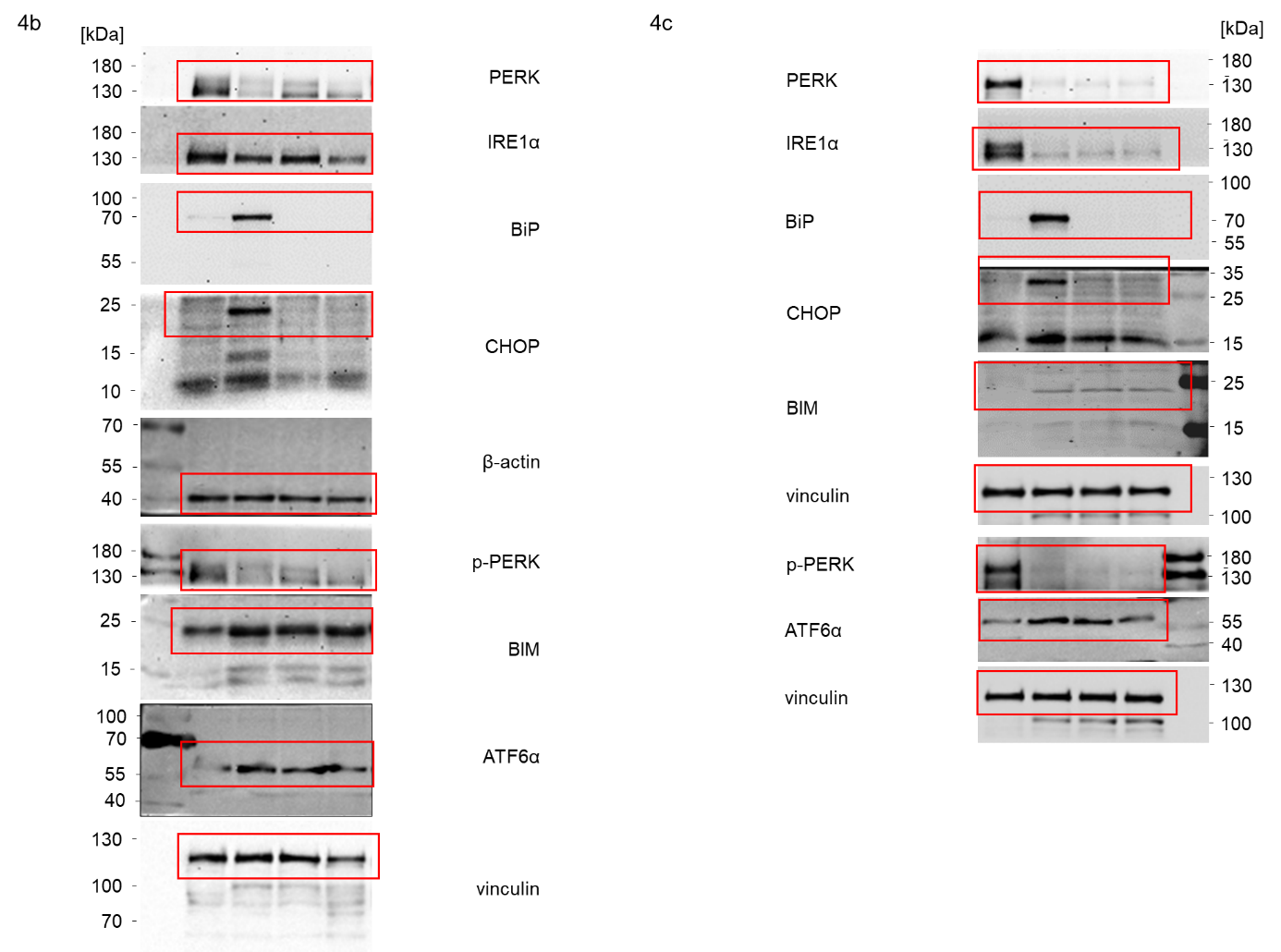


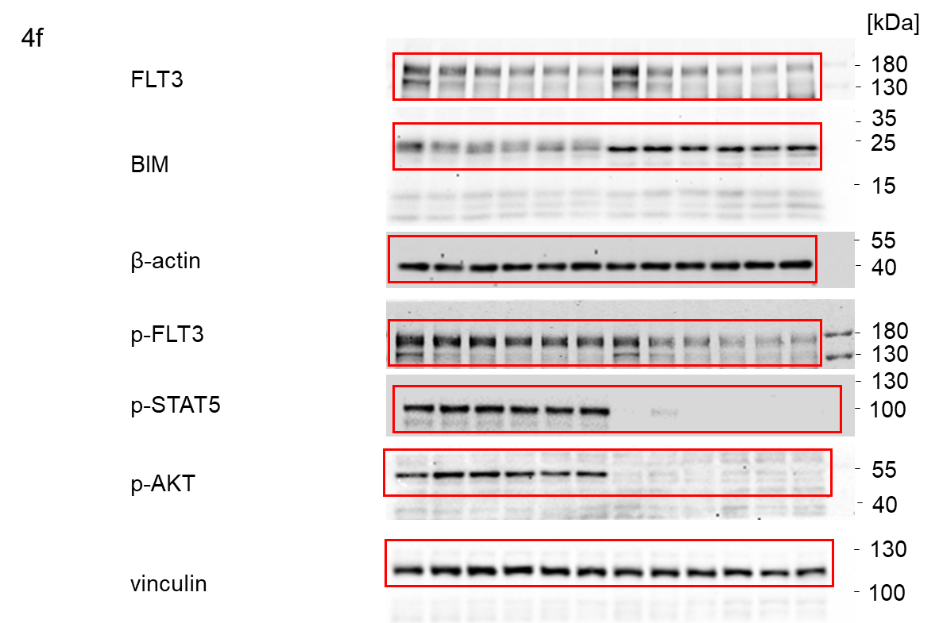


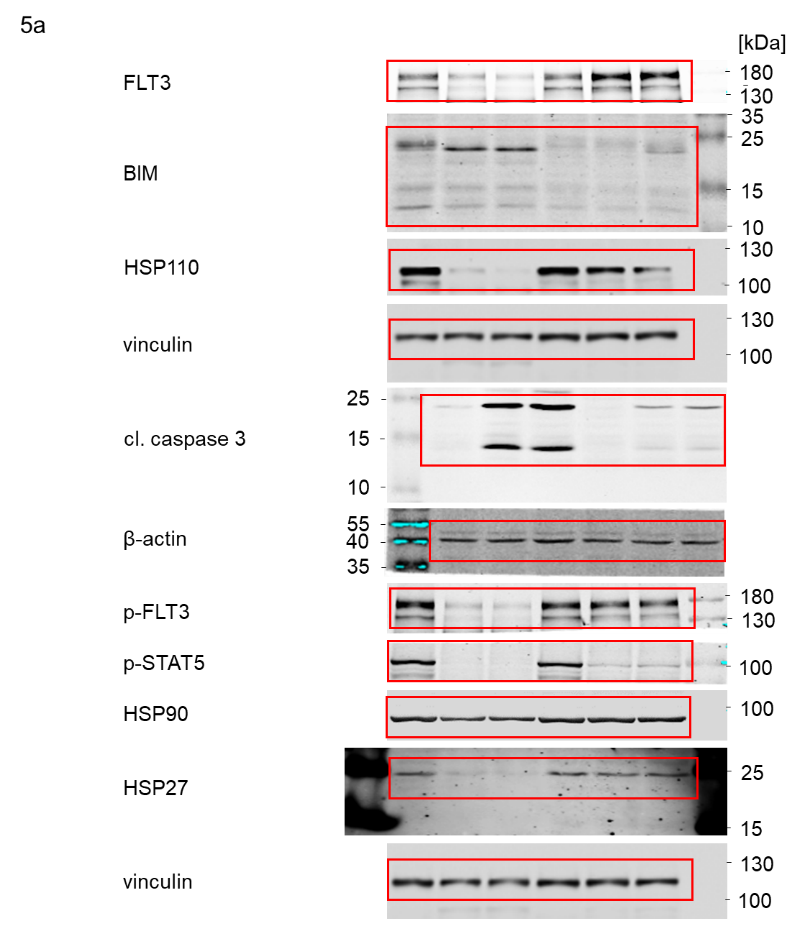


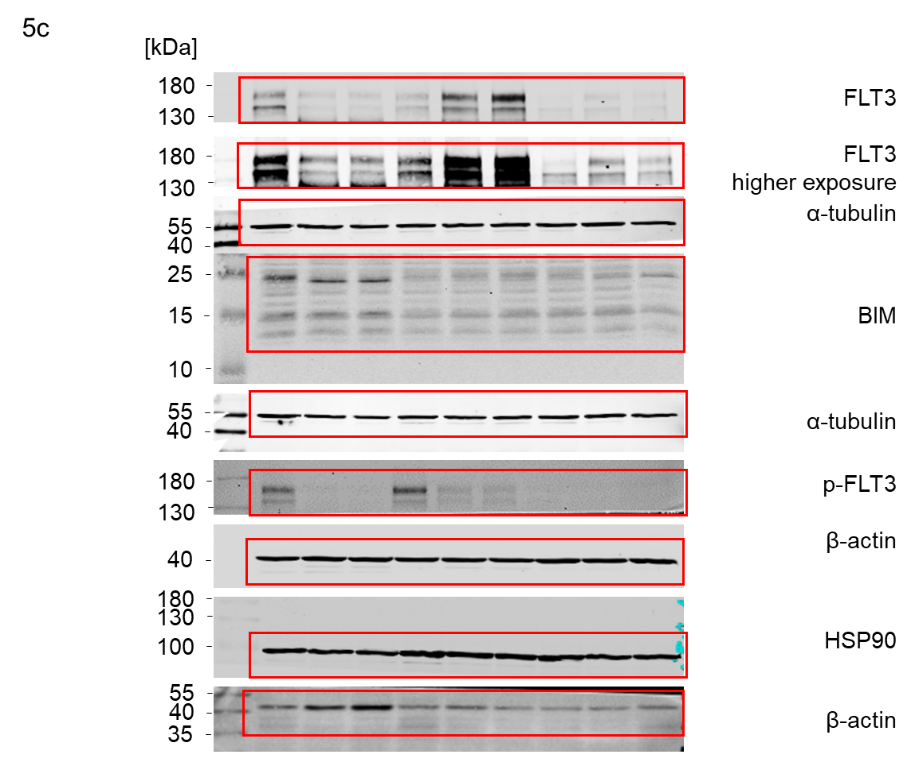


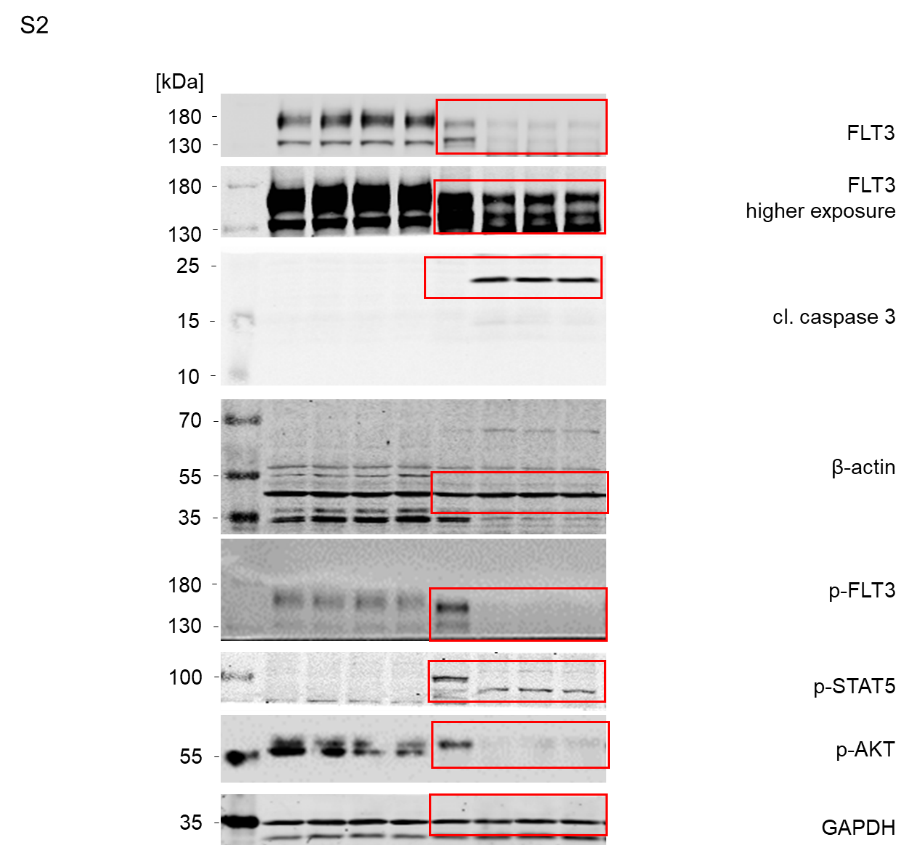


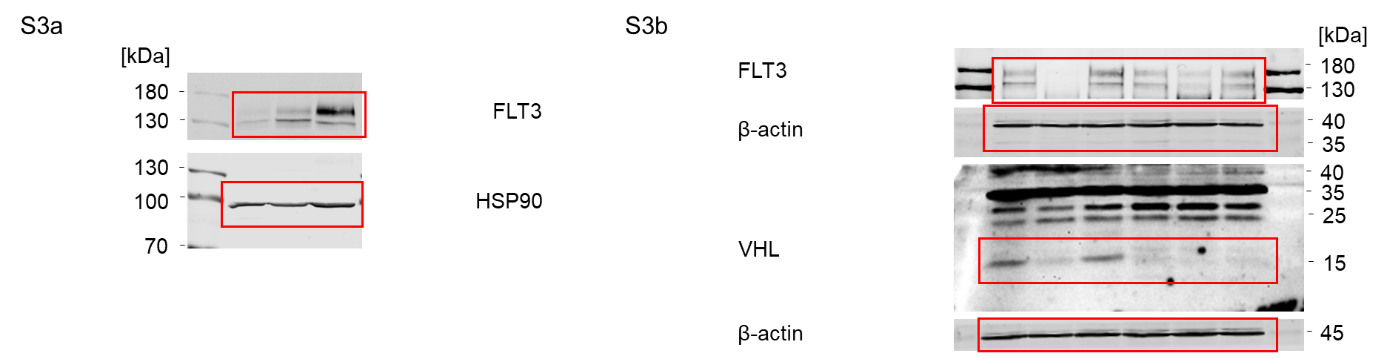


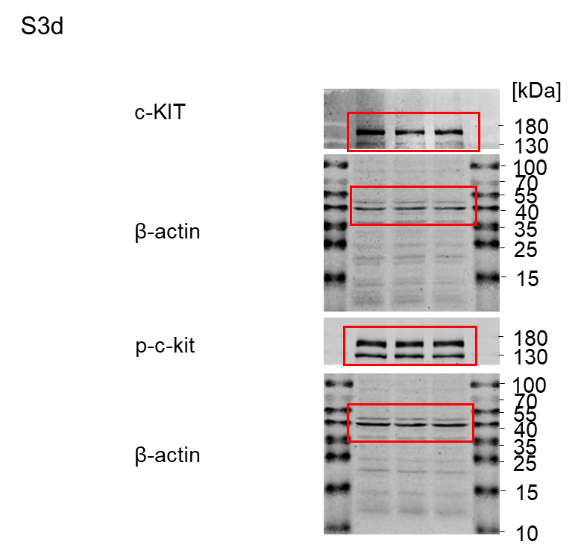


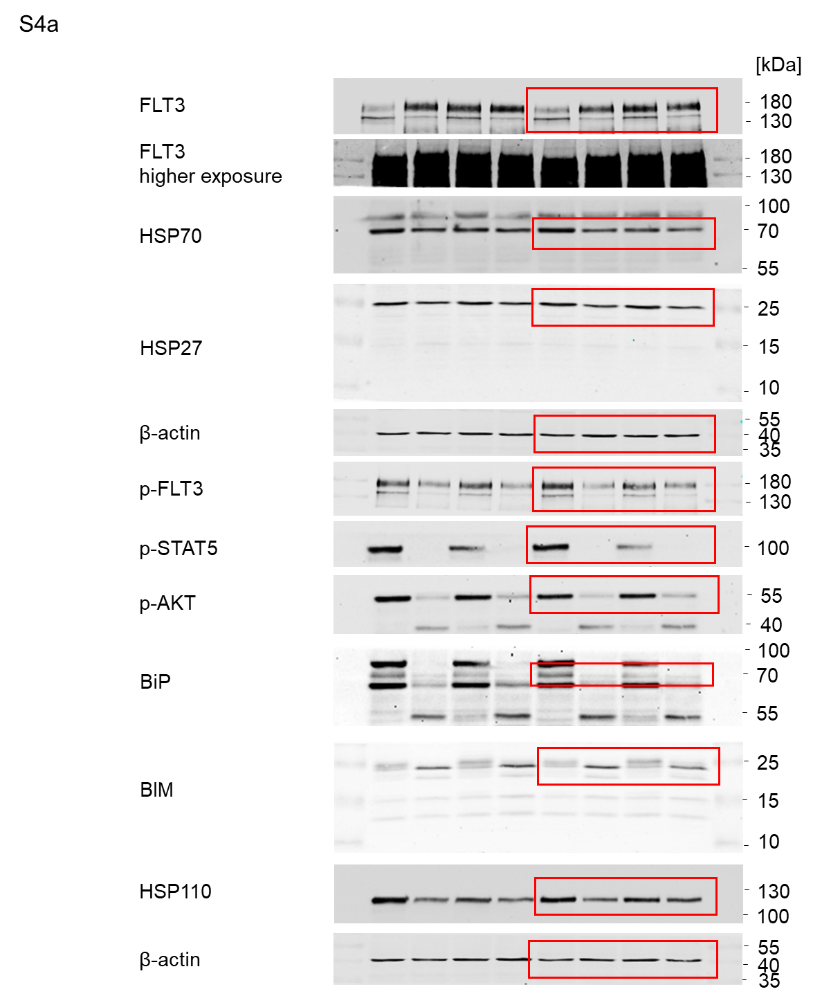


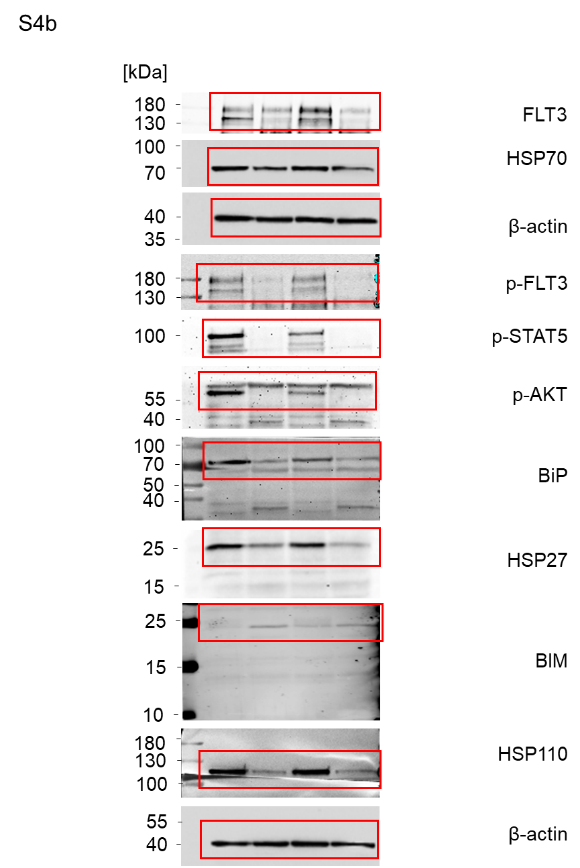

Supplement: Supplementary file 4 — Immunoblots - Original Files [file 41375_2024_2405_MOESM4_ESM.docx]
